# REFRESH: A New Approach to Modeling Dimensional Biases in Perceptual Similarity and Categorization

**DOI:** 10.1037/rev0000310

**Published:** 2021-09-13

**Authors:** Adam N. Sanborn, Katherine Heller, Joseph L. Austerweil, Nick Chater

**Affiliations:** 1 Department of Psychology, University of Warwick; 2 Department of Statistical Science, Duke University; 3 Department of Psychology, University of Wisconsin-Madison; 4 Warwick Business School, University of Warwick

**Keywords:** categorization, separable dimensions, family resemblance, Bayesian models

## Abstract

Much categorization behavior can be explained by family resemblance: New items are classified by comparison with previously learned exemplars. However, categorization behavior also shows a variety of dimensional biases, where the underlying space has so-called “separable” dimensions: Ease of learning categories depends on how the stimuli align with the separable dimensions of the space. For example, if a set of objects of various sizes and colors can be accurately categorized using a single separable dimension (e.g., size), then category learning will be fast, while if the category is determined by both dimensions, learning will be slow. To capture these dimensional biases, almost all models of categorization supplement family resemblance with either rule-based systems or selective attention to separable dimensions. But these models do not explain how separable dimensions initially arise; they are presumed to be unexplained psychological primitives. We develop, instead, a pure family resemblance version of the Rational Model of Categorization (RMC), which we term the Rational Exclusively Family RESemblance Hierarchy (REFRESH), which does not presuppose any separable dimensions in the space of stimuli. REFRESH infers how the stimuli are clustered and uses a hierarchical prior to learn expectations about the variability of clusters across categories. We first demonstrate the dimensional alignment of natural-category features and then show how through a lifetime of categorization experience REFRESH will learn prior expectations that clusters of stimuli will align with separable dimensions. REFRESH captures the key dimensional biases and also explains their stimulus-dependence and how they are learned and develop.

Categorization is central to the ability to encode knowledge, make inferences, and use language; and mastering huge numbers of flexible and complex categories is surely fundamental to human intelligence. How are categories represented and learned? Initially, researchers attempted to describe human categorization with logical combinations of rules: Objects were category members if and only if they satisfied a set of rigid constraints and objects that did not satisfy these constraints were left outside the category (Bourne, 1970; Bruner et al., 1956; Hull, 1920; Katz & Postal, 1964; Neisser & Weene, 1962). For example, a bus needs to have, among other things, wheels and space for passengers; anything not satisfying these criteria is not a bus. Category learning is then a matter of hypothesizing and testing logical combinations of rules (Shepard et al., 1961).

Though attractively simple, these rule-based descriptions of human category representations are too restrictive, as Wittgenstein (1953) argued. Even the apparently innocuous category of games turns out to have astonishing variety: card games, board games, word games, playground games, competitive versus cooperative games, individual and team games, and there is no rule that separates games from nongames. Moreover, the category has no clear boundary—for example, there is no sharp distinction between games and sports, or between games and pastimes (Sudoku, crosswords, solitaire); and the category continually grows and changes, and now includes the huge variety of computer games. Instead, games have a certain *family resemblance*—they appear similar to other games. Empirical evidence supports this intuition: the all-or-none nature of the simple rule-based categories was shown to be a poor fit to people’s representations, as participants instead have graded category representations in which objects have a better or worse claim to category membership (Rosch, 1973).

As a result, family resemblance is a critical element of many modern models of categorization: Exemplar models (Medin & Schaffer, 1978; Nosofsky, 1986), prototype models (Reed, 1972), and models based on multiple prototypes (Anderson, 1991; Love et al., 2004; Rosseel, 2002; Vanpaemel & Storms, 2008) all employ this kind of graded category representation. In these models, category judgments of new objects are based on their resemblance or similarity to the category representations; categories can have graded boundaries and can extend and change in the light of new instances.

But despite its overall success, family resemblance alone has also been viewed as insufficient. Some properties of the items to be categorized appear to play a special role: They seem to serve as underlying dimensions in terms of which the stimuli are represented. For example, what appear to be natural dimensions of a perceptual stimulus, such as size and color, significantly affect learning in ways that go beyond family resemblance. Explanations of these effects have either returned to rule-based models, or augmented family resemblance models with additional mechanisms like dimensional attention. We term these effects *dimensional biases*.

One classic dimensional bias is what is known as the condensation versus filtration effect: People learn categories that can be separated along a single dimension (e.g., color) more easily than they can learn categories that can only be separated by a combination of dimensions (e.g., Kruschke, 1993). Because family resemblance in this task predicts that the categories that require a combination of dimensions to separate should be learned at least as fast as the categories that can be separated by a single dimension, this result is taken as evidence that separable dimensions play a key role in category representations. A second demonstration is the classic result of Shepard et al. (1961): Category structures that can be described by simpler rules are easier to learn. Underlying dimensions seem to have no special role in family resemblance accounts; thus, dimensional biases are typically taken as a challenge to pure family resemblance accounts.

These empirical demonstrations appear to show effects both of family resemblance and of rules/dimensions, and also complex dependencies on the stimuli used. Hence, modern models of categorization have distinct mechanisms to capture each. The models that begin with family resemblance, such as the prototype and exemplar models of categorization (Medin & Schaffer, 1978; Nosofsky, 1986; Reed, 1972), also include psychological distance calculations and attentional mechanisms that are sensitive to separable dimensions, in order to explain dimensional biases. From the opposite starting point, modern rule-based models use separable dimensions as psychological primitives; they average a large number of rule-based structures or quickly switch between rules, to achieve the graded category structure considered the hallmark of family resemblance models (Feldman, 2000, 2006; Goodman et al., 2008; Goodwin & Johnson-Laird, 2013; Navarro, 2006; Nosofsky, Palmeri, et al., 1994; Shepard, 1987; Vigo, 2009). Finally, models using hybrid representations have two separate systems to produce rule-based and family resemblance behavior (Ashby et al., 1998).

These accounts capture the classic dimensional biases by design, but they are limited in a fundamental way. Categorization models mainly leave unaddressed the difficult problem of how these separable dimensions are actually learned, while the handful of models that do learn these dimensions (e.g., Colunga & Smith, 2005) are not able to capture all of the classic dimensional biases.

Here we take a very different, and potentially more unified approach. We model separable dimensions not as representational primitives. Instead, we model dimensional biases as the result of learning about the structure of the environment. In particular, we aim to show that the structure of many real-world categories aligns with the separable dimensions of the psychological space of stimuli. But the statistical properties of stimulus dimensions and their distribution across natural categories are not uniform throughout psychological space, which results in stimulus-dependence and related contextual effects, as outlined below. Specifically, we propose a Rational Model of Categorization (RMC), based exclusively on family resemblance, but one that is capable of capturing the complex and context-dependent structures found in real-world categories. In this model, context-dependent separable dimensions are a consequence of learning real-world categories, and thus, they are not presupposed.

In the following, first we outline the challenge of capturing human categorization performance with a pure family resemblance model. To do so, we describe the classic dimensional biases, why these biases depend strongly on the stimuli used, and the evidence that these biases are learned. Next, we review current models of categorization and discuss where they succeed and where they fall short in producing and learning dimensional biases. To illustrate our argument, we develop a computational-level model that is a pure family resemblance version of the RMC (Anderson, 1991), augmented with a hierarchical prior. We call this the Rational Exclusively Family RESemblance Hierarchy (REFRESH). The RMC works by inferring which objects should be clustered together. Crucially, REFRESH learns more than the specific clusters of stimuli that it observed, but also the types of cluster structures observed across categories in the environment. That is, it learns what sort of clusters should be expected: What cluster covariances tend to occur across categories. Next, we analyze the statistics of natural-category features, showing that the dispersion of these categories tends to align with features that researchers have found to be separable, such as shape and color. After the experience with categories with these types of statistics, REFRESH produces classic dimensional biases, as well as the stimulus dependence of these effects—all seven types of dimensional bias effects we describe below and in Table 1. Finally, we discuss the limitations of, and possible extensions to, our approach.[Table-anchor tbl1]

## Dimensional Biases

**Figure 1 fig1:**
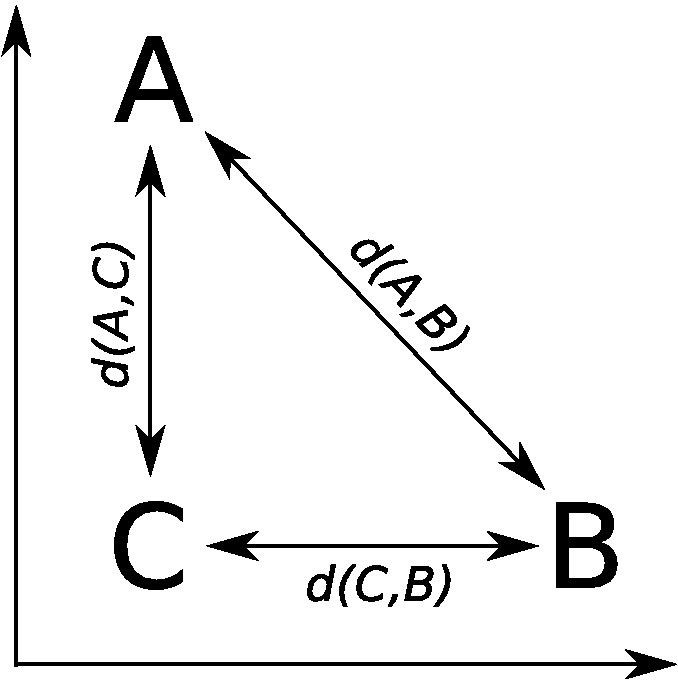
Example of Stimuli That Can Violate the Triangle Inequality *Note*. A, B, and C are stimuli within a psychological space defined by separable dimensions on the horizontal and vertical axes. If the distance between A and B, *d*(*A*, *B*), exceeds the combined distance *d*(*A*, *C*) + *d*(*C*, *B*) then it is not possible to represent the stimuli within this two-dimensional space.

There are several classic empirical demonstrations of dimensional biases, which are summarized in Table 1. Family resemblance models have at their heart the notion of similarity, which is commonly operationalized as the distance between stimuli in a mental space. However, for some dimensions, distance in a mental space is inadequate to explain similarity judgments because similarity judgments can violate the triangle inequality: The property that the greatest similarity or shortest distance is a straight line within the mental space. For example, assume that there are three stimuli, A, B, and C that have particular values on two separable dimensions as in Figure 1. If the stimuli have the following psychological distances, *d*(*A*, *B*) = 10, *d*(*A*, *C*) = 1, and *d*(*C*, *B*) = 1, then they cannot be represented within this space: The direct distance *d*(*A*, *B*) implies that A and B are far apart, while the path through stimulus C, *d*(*A*, *C*) + *d*(*C*, *B*) = 2, implies that A and B are close together. These triangle inequality violations occur if stimuli are aligned with the separable dimensions of the space as in Figure 1: If stimulus C matches both A and B on different separable dimensions while stimulus B mismatches A on both separable dimensions. This classic dimensional bias has been shown for a number of pairs of dimensions and is a strong argument against pure family resemblance models in which similarity is based on distance in a psychological space (Dunn, 1983; Soto & Wasserman, 2010; Tversky & Gati, 1982).[Fig-anchor fig1]

**Figure 2 fig2:**
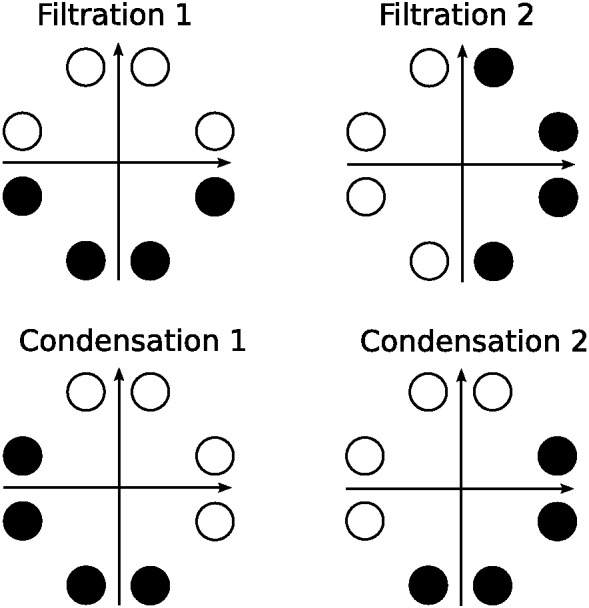
The Four Category Structures From Kruschke (1993) *Note*. The vertical and horizontal dimensions specify the height of a rectangle and the vertical position of a line within that rectangle, respectively. Each circle that is the same color belongs to the same category, and the vertical or horizontal distance between neighboring circles that differ on one dimension is assumed to be one unit. Adapted with permission from Kruschke (1993, p. 13).

Another classic dimensional bias is the difference between filtration and condensation categories. Filtration categories, or categories that can be separated using one dimension, are easier to learn than condensation categories, or categories that require two dimensions to separate (Garner, 1974; Gottwald & Garner, 1972, 1975; Kemler & Smith, 1978). A nice demonstration of the relative ease of learning filtration and condensation category structures was given by Kruschke (1993), who compared accuracy of learning the category structures shown in Figure 2. For these category structures, pure family resemblance models would predict the condensation categories are easier to learn because the two categories in the condensation condition are further apart than they are in the filtration condition. Despite this, participants found the filtration categories easier to learn.[Fig-anchor fig2]

**Figure 3 fig3:**
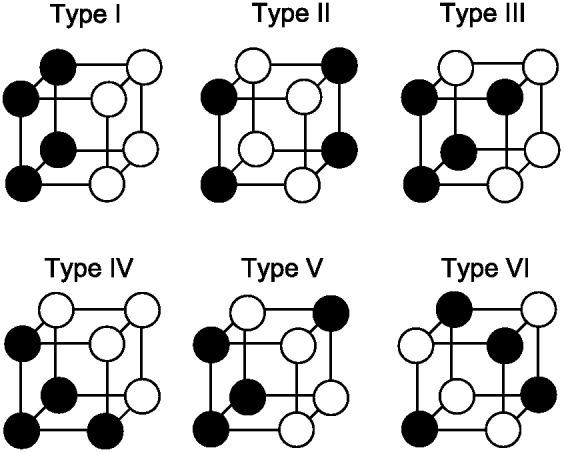
The Six Shepard et al. (1961) Category Structures *Note*. The three lines map onto three stimulus dimensions, such as the shape, color, and size of the stimulus if the dimensions are separable. Each circle that is the same color belongs to the same category, and the vertical and horizontal distances between neighboring circles are assumed to be one unit.

The third classic dimensional bias comes from the category learning experiments of Shepard et al. (1961, abbreviated here as SHJ). This work introduced six canonical category structures, shown in Figure 3, which comprise all the possible ways to divide the eight binary dimensional stimuli into two equal-sized classes, ignoring the mapping of the dimensions of the category structure onto the physical dimensions of the stimuli and the mapping of labels to categories. They collected same-different identification judgments for all pairs of stimuli, and family resemblance predicted a certain ordering of ease-of-learning: Type I categories should be easiest, then Types III–V, then Type II, and finally Type VI. However, participants learned Type II more quickly than Types III–V, which was evidence for the importance of separable dimensions in category representation: The Type II advantage occurred because one dimension can be ignored during learning, while Types III–V required participants to use all three dimensions to categorize the stimuli. The SHJ results are an example of an even more complex dimensional bias than that displayed in the filtration and condensation experiments. The filtration category structures could be perfectly divided along a single separable dimension, but the Type II categories required two separable dimensions to divide without error.[Fig-anchor fig3]

### Dimensional Biases Are Stimulus Dependent

While models generally treat separable dimensions as interchangeable, people’s degree of dimensional bias does depend on how the category structure is mapped to the physical dimensions of the stimuli. An obvious starting point for demonstrating this is the distinction between separable and integral dimensions (Garner, 1974). Size and color are an example of a pair of separable dimensions, because one dimension can be easily ignored when making judgments about the other. Integral dimensions, such as the hue and saturation of color, however, cause interference when making a judgment based on only one of the dimensions. This distinction between separable and integral dimensions applies to pairs of dimensions rather than to single dimensions. For example, hue and size are separable, but hue and saturation are integral. Further complicating the picture, the separable-integral distinction appears to be continuous rather than binary: Many pairs of dimensions seem to lie in-between these extremes (Soto & Wasserman, 2010; Tversky & Gati, 1982).

The classic dimensional biases depend on whether the dimensions are separable or integral. The advantage of filtration categories over condensation categories is found with separable dimensions and reverses when integral dimensions are used (Gottwald & Garner, 1975). The SHJ results also depend on whether the dimensions are separable and integral. The Type II problem is easier to learn than Type IV for separable dimensions but is not easier to learn for integral dimensions (Nosofsky & Palmeri, 1996). In both of these examples, using integral dimensions removes the dimensional bias, and the experiments using integral dimensions are instead well described by pure family resemblance. An additional difference between separable and integral dimensions was found by Soto et al. (2015), who showed that in biconditional discrimination (shown in Figure 16A, and which is essentially the same as learning only the upper half of the Type II problem in Figure 3), the correct responses were learned more quickly with integral dimensions than with separable dimensions.

Other work has shown that one of the classic dimensional biases is not found for all sets of separable dimensions. Love and Markman (2003) investigated the SHJ Type II and Type IV category structures using various mappings of the category dimensions to the physical dimensions of shape, size, and color. All pairs of these physical dimensions are separable, but only when Type II problems were mapped so that size and color were relevant, and shape was irrelevant for classification was the Type II problem reliably easier to learn than the Type IV problem. Kurtz et al. (2012) also found a dimensional dependence of the Type II advantage, and in another experiment demonstrated that there are stimulus dimensions that are separable but show no Type II advantage whatsoever. In this experiment, participants were able to quickly learn the single-dimensional Type I problem while not showing any Type II advantage. These complex dependencies of the Type II advantage on the stimulus dimensions have yet to be completely explained.

Existing models of categorization, as reviewed below, can make allowances for qualitative and sometimes quantitative distinctions between integral and separable stimuli. They can account for the differences between separable and integral dimensions in condensation versus filtration and SHJ tasks, and some models can also account for the finer-grained details in stimulus dependence, such as the SHJ Type II problem being easier to learn than Type IV for some sets of separable dimensions but not others. But these models do not generally explain how these dependencies are learned.

### The Role of Learning in Dimensional Biases

**Figure 4 fig4:**
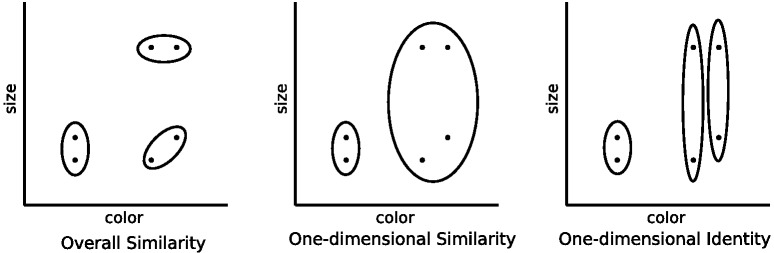
Experiment 2 of Smith (1989) *Note*. In a free categorization task, the stimuli marked by dots were grouped by participants. Stimuli that were grouped together are circled. The three plots show the three critical partitions. Adapted with permission from Smith (1989, p. 137).

One strand of evidence showing that dimensional biases are learned over development comes from free classification studies. In these experiments, participants are asked to group stimuli together as they see fit, and are usually allowed to use as many groups as they would like. This type of task allows for the size of the dimensional bias to be directly assessed: Do participants group together objects that match on dimensions but have low family resemblance, or do they group together objects that mismatch on dimensions but have high family resemblance? Examples of different types of grouping that reflect pure family resemblance (i.e., overall similarity), pure dimensional matching (i.e., one-dimensional identity), and both family resemblance and dimensional matching (i.e., one-dimensional similarity) are shown in Figure 4.[Fig-anchor fig4]

With separable dimensions such as size and color, adults’ free classifications show larger dimensional biases than those of children (Smith & Kemler, 1978). This developmental transition appears to be gradual. Children will group the stimuli according to family resemblance, but as the age of the participants increases the dimensional match becomes more and more important. Adults almost always produce dimensional matching (Smith, 1989). Other research has shown that for adults, an integral dimension can be trained to be more separable (Soto & Ashby, 2015). For example, color experts show more separability with color dimensions (Burns & Shepp, 1988) and participants trained on hue and saturation aligned categories show behavior that suggests that these dimensions become separated through training (Goldstone, 1994).

Dimensional biases in the SHJ task also increase during development. For Type I problems, 3-year-old children struggled, but 5- and 8-year-old children accomplished this task as well as adults do (Minda et al., 2008), which demonstrates that the 3-year olds were not able to use single-dimensional matches. The deficit in Type I problems was not a result of 3-year-old children not understanding the task: they were as good as adults at the Type IV problems. Echoing the dissociation between Type II and Type I performance found by Kurtz et al. (2012) in adults, 3-, 5-, and 8-year olds were all worse than adults at learning Type II categories, despite showing equivalent performance on other category structures (Minda et al., 2008). This suggests that the ability to use single-dimensional matches is learned and precedes the ability to use matches of conjunctions of dimensions, which develops later.

These developmental trajectories suggest that learning plays a role in dimensional development. Similar effects such as the shape bias for extending words to novel objects with the same shape have been explained as reflecting the structure of real-world categories (Samuelson & Smith, 1999). For example, some artifacts, like bowls or pencils, are categories that vary widely in color or material but typically vary less in their shape (Rosch et al., 1976). In addition, shape bias training studies have shown that children trained on named categories organized by shape were able to learn nouns faster outside of the laboratory than children not given this training (Smith et al., 2002) While this is a different dimensional bias than those considered above, it does suggest the possibility that the developmental changes in dimensional biases are the result of learning.

## Review of Models of Categorization

Here we briefly review a variety of existing models of categorization that all apply to incremental category learning experiments, grouping them into rule-based, family resemblance, and hybrid models. The RMC and hierarchical rational models are also reviewed, but in more depth as they form the basis for the new model we introduce in the next section. For all of these existing models, we discuss how well they can account for the classic dimensional biases and the stimulus dependence of these biases, and to what extent they can explain the learning of dimensional biases. A summary of the discussion below is presented in Table 2.[Table-anchor tbl2]

### Rule-Based Models

Rule-based models of categorization comprise some of the earliest descriptions of how categories are constructed (Bourne, 1970; Bruner et al., 1956; Katz & Postal, 1964; Neisser & Weene, 1962). While neglected for a long period of time due to the finding that category representations are graded (Rosch, 1973), later work has shown that uncertainty about rules can produce graded representations. The seminal work of Shepard (1987) demonstrated that if there is uncertainty about the extent of all-or-none categories, then averaging over these possible all-or-none categories will produce graded generalization. A similar approach has recently been used to produce graded category structures from rule-based representations: The all-or-none nature of each individual rule is smoothed out by averaging over a set of rules (Feldman, 2006; Goodman et al., 2008; Shepard, 1987; Tenenbaum & Griffiths, 2001b).

RULEX (Nosofsky, Palmeri, et al., 1994; Nosofsky & Palmeri, 1998) is another rule-based approach that starts with rules and later acquires exceptions: One-dimensional rules first, then conjunctive rules, and lastly exceptions if they are required. RULEX has been successful in matching the initial category judgments of participants, which are more rule-like than family resemblance models with selective attention predict (Nosofsky, Palmeri, et al., 1994). A bias toward single-dimensional rules over conjunctions is a straightforward route to producing the condensation versus filtration effects. Rule-based models can also easily produce violations of the triangle inequality (Tversky & Gati, 1982).

A more recent resurgence in rule-based representations has followed from the introduction by Feldman (2000) of an expanded set of SHJ problems that allowed unequal numbers of positive and negative examples, as well as additional dimensions. Participants’ ease of learning these concepts was found to correlate well with the minimum Boolean complexity of the concepts, but further research has demonstrated that this correlation was not perfect for the classic SHJ problems. In particular, Type II and Type III SHJ problems both have the same Boolean complexity (Kemp, 2012; Mathy & Bradmetz, 2003; Vigo, 2006), so later rule-based models have introduced new complexity measures (e.g., Feldman, 2006) that can match the empirical learnability advantage of Type II over Type III (Goodman et al., 2008; Goodwin & Johnson-Laird, 2011; Vigo, 2009).

Despite their advantages in predicting people’s learning of complex category structures, rule-based models do not always produce the stimulus dependence of dimensional biases and do not explain the role of learning in acquiring dimensional biases. Some rule-based models do include mechanisms for increasing or decreasing the use of rules based on experience (Feldman, 2006; Nosofsky, Palmeri, et al., 1994) or even for developing new primitives (Goodman et al., 2008). As a result, rules potentially could explain the SHJ Type II advantage dependencies and the development of latent dimensions, but it remains to be seen whether rule-based models can be modified to do so while explaining other human data. More importantly, these models cannot pick new dimensions as primitives in a continuous space, meaning that they cannot perform dimensional learning (Goodman et al., 2008). Also, effects found with integral dimensions are difficult to explain with rule-based models as they more naturally describe effects found with separable dimensions.

### Family Resemblance Models

Graded category structures motivated the development of family resemblance models of categorization (Rosch, 1973). Family resemblance can be implemented in a variety of ways, including both the classic prototype and exemplar models (Medin & Schaffer, 1978; Nosofsky, 1986; Reed, 1972). Other models interpolate between prototypes and exemplars by using representations that combine stimuli within the same category into one or more clusters (Anderson, 1991; Love et al., 2004; Rosseel, 2002; Vanpaemel & Storms, 2008). However, classic dimensional biases such as the condensation versus filtration results and the ordering of the SHJ problems have been used to argue that family resemblance representations alone are not sufficient to fully describe human categorization behavior. An additional mechanism is needed to produce dimensional biases, and as a result, models of family resemblance also have incorporated separable dimensions into their measures of similarity and distance.

**Figure 5 fig5:**
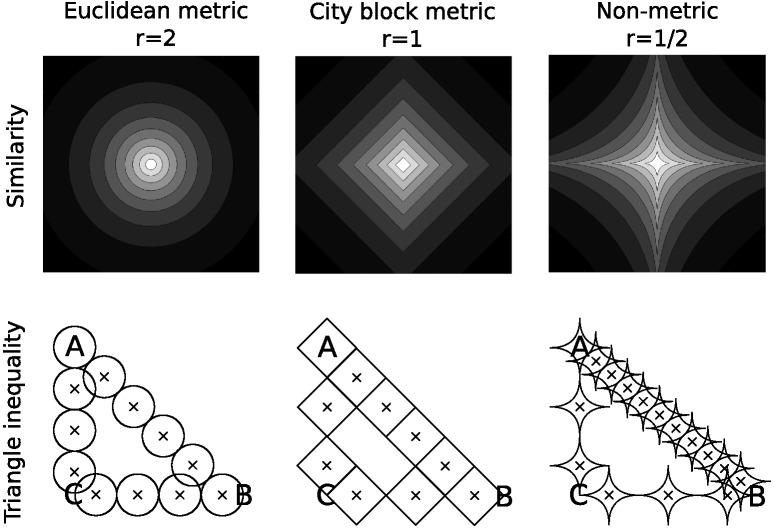
Illustrations of the Effect on Similarities and the Triangle Inequality for Different Values of the Minkowski Distance Metric Parameter r *Note*. The top row shows contour plots depicting the predicted similarity of a central stimulus to stimuli positioned at each point in the plot. The bottom row compares the distance along both a direct path (A to B) and an indirect path (though C) in terms of distance units for each value of *r*. Each distance unit is marked by an “×.” This illustrates why *r* < 1 is necessary for violations of the triangle inequality.

There are two common ways to incorporate separable dimensions into family resemblance models, both of which have been built into the calculation of distance between stimuli within the psychological space. Within many models, the distance between stimulus *x* and stimulus *y* is determined by a weighted Minkowski distance metric,

$$d={(\sum_{i}w_{i}(x_{i}-y_{i}{)}^{r})}^{1/r},$$ 1

where *w*_*i*_ is the weight assigned to each separable or integral dimension *i*, ∑_*i*_*w*_*i*_ = 1, and *r* is an exponent which determines the distance metric (Nosofsky, 1986; Torgerson, 1958). Figure 5 shows how this weighted Minkowski metric changes the distance calculation by showing curves of equal similarity around a central stimulus, where similarity is a monotonic transformation of distance. The difference in integral and separable dimensions is partially produced by changing the exponent *r*: A value of *r* = 2 (i.e., the Euclidean metric) is used for integral dimensions and a value of *r* = 1 (i.e., the city-block metric) is used for separable dimensions. The integral dimensions when *r* = 2 are not identifiable from the similarity curves: If the axes were rotated the exact same similarity curves could also be produced using the rotated axes. This is no longer the case for separable dimensions when *r* = 1: The separable dimensions are those that align with the “corners” of the similarity curves and are easily identifiable from these curves.[Fig-anchor fig5]

However, despite *r* = 1 often being used to describe separable dimensions, it cannot account for violations of the triangle inequality. In order to do so, values of *r* < 1 such as *r* = 1/2 are needed to fit the data. This can be seen in the bottom row of Figure 5. For both *r* = 2 and *r* = 1 the direct distance (as measured by the number of shapes) between stimuli A and B is not longer than a detour through stimulus C. However, for *r* = 1/2 the direct distance is longer than a detour through stimulus C, violating the triangle inequality. Because of this, values of *r* < 1 mean that the Equation 1 is no longer interpretable as a distance metric, though it can still be used within these models to fit data (Nosofsky, 1984). However, even allowing for values of *r* < 1, no single value of *r* can explain why violations of the triangle inequality occur for stimuli that are easily distinguishable (Dunn, 1983; Soto & Wasserman, 2010; Tversky & Gati, 1982), but also that the Euclidean metric is the best fit for stimuli that are confusable (Nosofsky, 1986).

**Figure 6 fig6:**
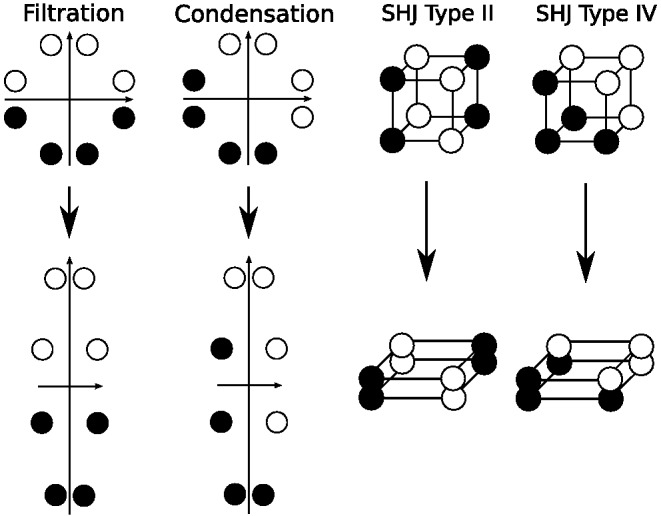
Illustrations of how Selective Attention Produces the Condensation Versus Filtration Effect and the SHJ Type II Advantage *Note*. The top row shows the key original conditions from these two experiments, as reproduced from Figures 2 and 3. The bottom row shows the effect of selective attention, which stretches and shrinks the dimensions of the psychological space to make stimuli more or less distinguishable. Selective attention better separates the categories for the filtration and SHJ Type II categories than it does for the other conditions, producing learnability advantages for the filtration and SHJ Type II categories.

The second way to incorporate separable dimensions in family resemblance models is to allow for selective attention to separable dimensions. This route involves adjusting each weight *w*_*i*_ in the weighted Minkowski metric for the separable dimensions, which in effect stretches or shrinks the distances in the psychological space along the separable dimensions, as demonstrated in Figure 6. As the stretching and shrinking of the psychological space happen along the separable dimensions, it can be used to effectively separate the two categories in the filtration conditions, making them easier to distinguish. For the condensation categories, because stretching or shrinking can only operate along the separable dimensions, performance does not improve as much, and this limitation to stretching or shrinking has been demonstrated qualitatively as well (McKinley & Nosofsky, 1996). This explains the learning advantage in the filtration conditions. The same explanation applies to the SHJ Type II advantage, as selective attention separates the Type II categories better than it does the Type IV categories (Nosofsky, 1986). Models such as ALCOVE are able to learn how to effectively deploy selective attention during category learning (Kruschke, 1992).[Fig-anchor fig6]

Selective attention can produce some classic dimensional biases, and can also produce some kinds of stimulus-dependence of these biases. It can explain, and indeed it predicted that the SHJ Type II advantage would disappear for integral dimensions (Nosofsky & Palmeri, 1996). It does so by not allowing the weights *w*_*i*_ to be learned during the task. However, more complex parameterizations may be needed to explain the pairwise nature of separable and integral dimensions, why SHJ Type II performance is sometimes poor while Type I performance is excellent, and the developmental trajectory of the relative difficulty of the SHJ problems (Kurtz et al., 2012; Minda et al., 2008). Importantly, how family resemblance models learn which dimensions to apply selective attention to is what is most difficult to explain. Learning the best dimensions to attend to over the course of an experiment from the set of possible preferential dimensions has been well modeled (Kruschke, 1992, 1993; Nosofsky, Gluck, et al., 1994), as has the development of separable dimensions from a latent set of dimensions (Smith, 1989). What is missing in these models is an explanation of how the set of latent separable dimensions are learned.

### Hybrid Models

Of course, selective attention is not the only route for introducing dimensional biases into family resemblance models. Another way to do so is with hybrid models, such as ATRIUM (Erickson & Kruschke, 1998) and COVIS (Ashby et al., 1998), that combine the strengths of family resemblance and rule-based models into a single package. COVIS is an especially well-studied model which has two systems: A rule-based system and a family resemblance system that are assumed to be situated in different parts of the brain. COVIS has been successful in describing dissociations between learning of category types that depend on different systems, which we discuss in more detail in the Explaining Evidence for Multiple Systems with a Single System section below. These hybrid approaches can explain the classic dimensional biases, and potentially could explain the stimulus-dependence of dimensional biases by tying the availability of types of rules to different dimensions. Hybrid models have the potential to explain the differential development of SHJ Type I and II problems, if rule primitives are built in (Minda et al., 2008). However, it is not clear how these models could be extended to explain the learning of those primitives.

### The Rational Model of Categorization

The RMC (Anderson, 1991) casts categorization as inference about the unobserved aspects of stimuli, such as the category labels that should be assigned to new, unlabeled stimuli. The RMC is a mixture model which can interpolate between an exemplar model and a prototype model—there could be one *cluster* that describes all the stimuli within each category, as in a prototype model, or there could be as many clusters as there are individual stimuli, as in an exemplar model. Instead of being restricted to a single cluster or a cluster for each previous stimulus, the mixture model has the flexibility to choose an intermediate number of clusters.

When determining the category label of a new stimulus, the statistical model underlying the RMC assumes that every possible assignment of stimuli to clusters is considered. Computing exact probabilities using this underlying statistical model is both computationally intractable and psychologically implausible, so to solve these issues Anderson (1991) developed a simple approximation as a core part of the model. This approximation assumed a single “history”: that every item was assigned to a single cluster, specifically the cluster that was most likely when that item was first observed. While this approximation is often accurate, later work used other tractable approximations which increase accuracy by representing multiple possible histories (Sanborn et al., 2010). We focus here on the computational-level model underlying the RMC, defined by Anderson (1991) and later shown to be equivalent to a well-known Bayesian nonparametric model in statistics (Neal, 1998), because it is used as a basis for REFRESH. We discuss approximations both below and in the Appendix.

When a new item is observed, the probability of assigning the new item to a cluster depends on both the prior probability of each cluster and the likelihood of each cluster. The prior probability of each cluster is based on a rich-get-richer process, the Chinese Restaurant Process (CRP), in which the prior probability is roughly proportional to the number of items already in the cluster, but with a small probability reserved for assigning the new item to a new cluster. This allows the RMC to have the flexibility to always increase the number of clusters as the mixture model observes more items. More formally, assume we call the cluster index of the *n*th stimulus *z*_*n*_, while the vector of the cluster indices of all previous stimuli is called **z_n−1_**. Then the prior is

$$P(z_{n}=k|z_{n-1})=\{\begin{array}{cc} \frac{M_{k}}{n\;-\;1\;+\;\alpha } & \text{if}M_{k}>0\;(\text{i.e.},\;k\text{is old})\\ \frac{\alpha }{n\;-\;1\;+\;\alpha } & \text{if}M_{k}=0\;(\text{i.e.},\;k\text{is new}), \end{array}$$ 2

where *M*_*k*_ is the number of objects assigned to cluster *k*, and α is called the dispersion parameter.^1^ Using Equation 2, the set of assignments **z_n−1_** is defined as a simple sequential stochastic process (Blackwell & MacQueen, 1973) in which the order of the observations is unimportant (Aldous, 1985).

In the RMC, the likelihood that a new item belongs to a cluster depends on how well the features of a new stimulus match those of the stimuli that are cluster members. The model additionally assumes that the features of the items within a cluster are independent of one another, so that there are no correlations between features. For binary features, such as category labels or binary perceptual features, a beta-binomial distribution with parameter β is used to model the likelihood that a binary feature arises from a particular cluster.

However, for continuous perceptual data like in the condensation versus filtration problems, the likelihood used for the perceptual features is Gaussian along each dimension, and there is a prior on both the mean and variance parameters of each Gaussian. More formally, the mean and variance of the Gaussian distribution for the *k*th cluster on the *d*th dimension are given by μ_*k*_^(*d*)^ and Σ_*k*_^(*d*)^respectively, where μ_*k*_^(*d*)^ is the *d*th element of the vector μ_*k*_ and Σ_*k*_^(*d*)^ is the *d*th diagonal element of the diagonal matrix Σ_*k*_. The prior for the mean was assumed to be Gaussian given the variance, and the prior for the variance was assumed to be an inverse-χ^2^ distribution

$$\mu_{k}^{\left(d\right)}\;\;∼\;\;N\;(\omega ,\Sigma_{k}^{\left(d\right)}/\lambda_{0})\Sigma_{k}^{\left(d\right)}\;∼\;\;\text{inverse-}\chi^{2}(a_{0},\;\sigma_{0}^{2}),$$ 3

where λ_0_ is the confidence in the prior mean, *a*_0_ is the confidence in the prior variance, σ_0_^2^ is the prior variance, and ω is the prior mean.

The RMC uses the probability of assigning a new item to each cluster to come to a category decision, along with the known category labels of the items already assigned to each cluster. This computation is a weighted sum, with the known category labels determining how likely the label is within each cluster, and each label likelihood is weighted by the probability of assigning the new item to that cluster. So, essentially the decision rule averages over the uncertainty of how well a new item fits each cluster, and how well a category label fits each cluster as well. A more formal definition of this process is given in the Appendix.

The likelihood distribution for the RMC assumes a fixed basis set of dimensions, which must align with the separable dimensions to produce dimensional biases. As a result, the RMC is able to produce some, though not all, of the classic dimensional biases. The condensation versus filtration results can be produced because the dimensions are aligned with the identifiable dimensions of the stimuli if the prior distribution for the variances is set to the correct values, as we demonstrate by matching the ordering of the conditions of the human data (see Figure 12A) with the model simulations (see Figure 12B). For this simulation, we used the original approximation to the model (Anderson, 1991), and found that the following RMC parameters qualitatively matched the human data: α = 1, σ_0_^2^ = 2.25, *a*_0_ = 1, λ_0_ = 1, β = 0.1, and ω equal to the average value of the stimuli on each dimension.

The ordering of the SHJ problems can also be produced using a discrete binary likelihood, but the correspondence of the model to the canonical ordering is parameter dependent and the parameters that produce this ordering are often not those that produce the best match to the overall accuracy level of human performance (Nosofsky, Gluck, et al., 1994; Sanborn et al., 2010), though it has been successful on occasion (Badham et al., 2017). The RMC for continuous data is unlikely to be able to produce violations of the triangle inequality because the probability of being a member of a cluster is Gaussian which corresponds to a Euclidean distance metric, as we discuss in the Appendix. Similarly, the RMC also cannot capture the pairwise nature of separable dimensions or dimensional learning. Because of its fixed prior, it does not seem possible for the RMC to produce the SHJ Type II advantage dependencies or SHJ Type I and II differential development. Finally, the RMC uses a fixed set of dimensions. So, it will struggle to explain how dimensional biases develop, and it cannot explain how new separable dimensions are learned.

### Hierarchical Rational Models

Previous work with computational-level Bayesian models has taken steps in a similar direction to those that we take in REFRESH, proposing hierarchical priors that can learn how to generalize, using either the RMC or the notion of consequential regions as a foundation (Shepard, 1987). Consequential regions are all-or-none neighborhoods in a psychological space that correspond to stimuli with a common outcome or consequence. When observing a new category, the size of the region along each dimension is uncertain, but there is prior knowledge of the distribution of sizes along each dimension that can be used. Navarro (2006) added priors along separable dimensions and showed that the model provided a computational account of selective attention, as this model was able to capture the condensation versus filtration dimensional bias. Austerweil and Griffiths (2010) extended this approach to allow the model to learn which kind of hypothesis space best applied to the stimuli: One in which the consequential regions produced the Euclidean similarity metrics associated with integral dimensions, or one in which they produced the city-block similarity metrics associated with separable dimensions (see also Austerweil et al., 2019). Soto et al. (2014) augmented this model to allow it to learn latent causes and thus explain compound generalization along both separable and integral dimensions.

Another set of models have used the RMC as a foundation. For example, the model of Kemp et al. (2007) learns the variability along particular dimensions for categories from experience, as the RMC does, and also includes the possibility of stimulus dependence in the types of variability that are learned. Another, developed by Salakhutdinov et al. (2012) for computer vision using low-level visual features such as pixels, learns both the variability along particular dimensions and the stimulus-dependence of the variability as well.

These models show how a hierarchical Bayesian approach would be able to produce the dimensional biases and the stimulus-dependence of dimensional biases, and they should be able to show the development of latent dimensions that are prespecified in the model. These hierarchical rational models however have at most, as in the case of Austerweil and Griffiths (2010), learned from a small number of prespecified dimensions and have not been extended to learn separable dimensions that are not prespecified.

## The Rational Exclusively Family Resemblance Hierarchy

Our new model is based on the RMC, but without any inbuilt separable dimensions. In our approach, which we term REFRESH, we modify the likelihood of the RMC so that it starts purely with resemblance, and not dimensions, and equip it with a hierarchical prior that allows dimensional biases to be emergent properties that arise from the family resemblance structure of the data. Our model is a computational-level model, though it has free parameters that we chose to match human data, and we explore possible algorithmic factors in a later section. In this section, we first give an intuition of the key features of the model followed by a more technical description of these features, and further technical details are given in the Appendix.

To create a pure family resemblance model, we start with the RMC, but assume that all the perceptual stimuli used in categorization experiments lie within a continuous space, even if the specification of the category structures can be done with binary features (e.g., the SHJ problems in Figure 3). This assumption mirrors that made by many other models of categorization (Ashby & Townsend, 1986; Kruschke, 1992; Nosofsky, 1986), and removes the necessity of identifying the separable dimensions of the stimuli in order to specify their discrete features.

A key step in creating a pure family resemblance model is to change the likelihood of the RMC. The RMC treats the likelihood of continuous perceptual stimuli as arising from the product of single-dimensional Gaussian distributions along each of the separable dimensions. Instead, our model assumes that the stimuli within a cluster are samples from a single multivariate Gaussian distribution. The usefulness of the multivariate Gaussian distribution for our purposes can be illustrated by comparing it to the weighted Minkowski metric in Equation 1. It is straightforward to show that the weighted Euclidean metric (with *r* = 2) is a monotonic transformation of a multivariate Gaussian distribution, one that has a diagonal covariance matrix, and the weight for a dimension is the inverse of the variance along that dimension (see Appendix). This means that a multivariate Gaussian distribution with a diagonal covariance matrix stretches or shrinks the stimulus space along a set of dimensions, just as selective attention does. As REFRESH is pitched at Marr’s computational level (Marr, 1982), it is complementary to an algorithmic account based on selective attention.

What multivariate Gaussian distributions add is that they are not confined to stretching or shrinking the space along a single prespecified set of dimensions. This is because the covariance matrix is allowed to be nondiagonal, and a nondiagonal covariance matrix is always diagonal with respect to some rotation of the dimensions of the space. This essentially means that a multivariate Gaussian distribution can implement a stretching or shrinking of the space along any rotation of the dimensions of the space, with that rotation being the one needed to make the covariance matrix diagonal. So, a multivariate Gaussian’s covariance matrix encodes both a set of dimensions as well as the amount of stretching or shrinking along those dimensions (see Appendix).

Interestingly, for the multivariate Gaussian, the dimensions along which the stimuli are represented—the dimensions that provide the coordinates of each stimulus—are irrelevant to the likelihood. The representing dimensions (i.e., the axes of the space) can be rotated in an infinite number of ways, and for each possible rotation there is a new parameterization of the multivariate Gaussian distribution that gives exactly the same probability to each and every stimulus as the original multivariate Gaussian did (see Appendix). As a result, the predictions of REFRESH are independent of the dimensions used to represent the stimuli, and even independent of whether the representing dimensions are fixed or are changing. Therefore, a careful analysis of algorithmic- or implementation-level concerns, as well as data from neuroscience, will be necessary to motivate the choice of representing dimensions.

**Figure 7 fig7:**
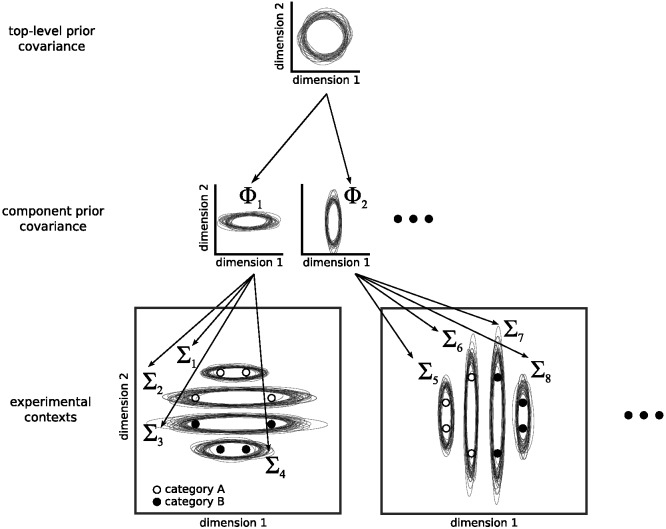
Schematic Illustration of the Rational Exclusively Family RESemblance Hierarchy Model *Note*. At the lowest level of the model’s hierarchy are experimental contexts, and here the two contexts are the two filtration category structures of Kruschke (1993, see Figure 2). In each context, the model’s task is to learn the category label (category A is open circles while category B is filled circles) of each stimulus. The model infers how each category should be partitioned into clusters, with each of the *K* clusters (with K a random variable) modeled by a multivariate Gaussian distribution with covariance parameter Σ_*k*_. To illustrate the model’s uncertainty for each Σ_*k*_, a number of sample covariance matrices are shown for each as iso-probability ellipses. At the next level up in the hierarchy, the model simultaneously learns to describe all of the Σ_*k*_ across contexts as a mixture of components. It learns the number of components, *J* (which is also a random variable), their weights, and the covariance matrix parameter Φ_*j*_ that determines the most likely covariance matrix of component *j*. Uncertainty about the covariance matrix for a new cluster (e.g., Σ_9_) is shown for each component by sample iso-probability ellipses. At the top of the hierarchy the model has a prior for the Φ_*j*_ covariance parameters, which is on average isotropic. Uncertainty about the covariance matrix parameter for a new component (e.g., Φ_3_) is shown by sample iso-probability ellipses.

A pure family resemblance version of the RMC will, of course, be unable to produce any of the classic dimensional biases because it does not have any preferred dimensions. To acquire such biases, we need to equip the model with a hierarchical prior that can learn the variability of clusters of stimuli across categories. We initially choose a commonly used multivariate prior for the covariance of a cluster *k*, Σ_*k*_: the multivariate generalization of the inverse-χ^2^ distribution, the inverse-Wishart (IW) distribution. An IW distribution is commonly used because, for a fixed number of dimensions, it is a single conjugate multivariate distribution that assigns a probability to each possible multivariate covariance matrix with that number of dimensions. This distribution is parameterized by the number of dimensions of the stimuli *D* as well as two free parameters: a multivariate covariance matrix parameter, Φ, and a degrees of freedom parameter, *v*. The most probable covariance matrix in the IW distribution is $\frac{\Phi }{v\;+\;D\;+\;1}$, and visualizations of covariance matrix samples drawn from IW priors are shown in Figure 7.[Fig-anchor fig7]

Using this prior gives the model flexibility to learn how stimuli vary across categories, but this prior is still inherently restricted in ways that do not allow it to produce dimensional biases. With this prior, the model can learn a particular kind of generalization bias within the space, so that the expected variability of stimuli in new categories is similar to what has been experienced in previously learned categories. However, the predicted similarity metrics will remain close to Euclidean (as will be demonstrated in the next section in Figure 11), so the model cannot capture the similarity metrics associated with separable dimensions.

Thus, in order to explain the dimensional biases, we need a more flexible formulation of the hierarchical prior. We do this in the same way that we provide flexibility to the clusters within a category: We turn this prior into an infinite mixture of different *components*, which effectively allows the prior to have multiple modes, with each mode belonging to a different component. A component is a higher level analog of a cluster: A cluster has a mean and a covariance matrix that describe the stimuli that are members of a cluster, while a component has a covariance parameter that roughly describes the covariances of the clusters that are members of the component.^2^ Figure 7 gives a schematic depiction of the hierarchical prior used in REFRESH. We can think of an item as being drawn from a cluster, which has a prior that is drawn from the set of components that have been used in the local context. This set of components used in the local context have themselves been drawn from a global distribution over possible components. The covariance parameter of each component finally has a prior that has no alignment with the separable dimensions of the space. This scheme of sharing information across clusters is a generalization of the unifying model developed in Griffiths et al. (2007).

More technically, for each component *j*, we define Φ_*j*_ to be the covariance parameter of that component and *v*_*j*_ to be that component’s degrees of freedom. Assuming that a cluster *k* has been assigned to component *j*,

$$\mu_{k}\;∼\;N(\omega ,\;\sigma_{r}^{2}I)\Sigma_{k}\;∼\;IW_{v_{j}}(\Phi_{j})\Phi_{j}\;∼\;IW_{v_{t}}(I)v_{j}\;∼\;TN(v_{t},\;v_{t}^{2}),$$ 4

where *I* is the identity matrix, and σ_*r*_^2^*I* is a scaled version of the identity matrix, meaning that both *I* and σ_*r*_^2^*I* are isotropic and not oriented toward any particular direction in the space. Each *v*_*j*_ has a truncated normal (TN) prior that is truncated from below at the number of dimensions of the stimulus, and has mean and standard deviation equal to the degrees of freedom of the top-level prior *v*_*t*_. Comparing Equation 3 with Equation 4, we can see that in addition to becoming multivariate and hierarchical, we have made the cluster mean vector μ_*k*_ independent of the cluster covariance matrix Σ_*k*_. We made this choice pragmatically as we found that assuming independence between means and covariances resulted in a better fit to human data.

As we did above for the vector of assignments of all items to clusters, **z**, we use a nonparametric prior over the vector of assignments of all clusters to components **u**. For **u**, we use a hierarchical CRP prior (Teh et al., 2006), which is an extension of the single-level CRP prior used for **z** to two levels. It corresponds to the intuition that while there are a variety of possible components that can apply to a cluster, there tends to be only one or a small number of components that apply within a particular experimental context. That is, a component that has already been used in an experimental context is more likely to be used for the next new cluster in the same experimental context. In the hierarchical CRP, there are two additional dispersion parameters: α_*c*_, which determines the probability of bringing an existing component into the current context, and α_*g*_, which determines the probability of creating a new component. The equation that governs these probabilities is described and given in the Appendix by Equation A9. Finally, for simplicity, we assume that there is no possibility of a mixed cluster as there is in the RMC, so that each cluster consists of items from a single category.

We fix a number of the parameters of REFRESH to particular values for consistency across simulations. The dispersion parameter for new clusters, α = 10, was set to a relatively high value to encourage new clusters, while the dispersion parameter for bringing a component into the current context, α_*c*_ = 0.001, was set to a relatively low value so as to encourage only one or a few components within an experimental context, which was necessary to fit empirical results. The dispersion parameter for new components across experimental contexts, α_*g*_ = 1, was set to an intermediate value so as to encourage a small number of components. The degrees of freedom, *v*_*t*_, which describes how certain we are about the top-level prior covariance matrix, as well as determining the mean and variance of the prior on each *v*_*j*_, was set to 30 to reflect moderate certainty in the initial isotropic prior distribution. The remaining model parameters were set according to the specifics of the simulation, as reported below, and summarized in Table A2. In all of the simulations of the model, results were either calculated exactly or approximated using particle filters or Gibbs sampling (depending on the application) using the number of samples that we believe would accurately reflect the underlying computational model and not the approximations themselves (see Appendix for details). We mainly investigate the computational-level model in the main text, though we consider the impacts of algorithmic-level approximations on the computational-level model in the Rational Process Models section below.

## Learning From the Statistics of Natural Categories

Some artifacts, like bowls or pencils, are categories of objects that vary widely in color or material but typically vary less in their shape (Rosch et al., 1976). However, categories of materials such as gold or wood often display a characteristic color while being less constrained as to the shapes and sizes that they take. There are, of course, categories that are constrained along two dimensions simultaneously, such as crayons, which have a characteristic shape and a common material but vary widely in color (Gershkoff-Stowe & Smith, 2004). These types of regularities have been found in the nouns that children learn first. An investigation into the statistics of the first 300 nouns learned by children, found in the MacArthur Communicative Development Inventory (MCDI; Fenson et al., 1994), was made by Samuelson and Smith (1999), who asked adults how these categories were organized. The results showed that solid objects tend to have a fixed shape and vary along other dimensions, while nonsolid objects vary in shape but tend to be of fixed material, a regularity that could drive later generalization of category labels to new stimuli.

Along these same lines but more generally, Shepard (1987, 1991) hypothesized a relationship between the variability of the dimensions of categories, or more precisely the variability of the dimensions of consequential regions, which are sets of stimuli that share the same consequence. The hypothesis was that the natural statistics of how dimensions vary across categories should determine whether dimensions are integral or separable. Dimensions with positively correlated variability should be integral, whereas dimensions with uncorrelated variability should be separable. For example, categories that were highly variable along the hue dimension would also be highly variable along the chroma dimension, while categories with low variability along the hue dimension would also have low variability along the chroma dimension. Shepard (1987) showed that integrating over consequential regions with correlated variability produced Euclidean similarity metrics, as is empirically found for integral dimensions. However, for separable dimensions variability along these dimensions was hypothesized to be uncorrelated, so for example, a category highly variable along the hue dimension could have either high or low variability along a shape dimension. Uncorrelated variability produced city-block similarity metrics in Shepard (1987) analysis, as is often empirically found for separable dimensions.

Both the regularities in the nouns first learned by children and Shepard (1987, 1991) hypothesis inspired the development of REFRESH, but to our knowledge the hypothesized correlations in the variability of categories along separable and integral dimensions have not been investigated empirically. To obtain objective quantitative evidence for these regularities, we examined the statistics of natural images using a database produced by Rosenthal et al. (2018) of images from the internet containing salient objects.^3^

The strength of this method for determining natural-category variability is that it does not require subjective judgment and it produces precise values for each image along each dimension, even for integral dimensions which cannot be isolated by human participants. The weakness is that these photographs were downloaded from the internet, and so have biases related to how they were produced, such as that people prefer to observe objects from “canonical perspectives” (Palmer et al., 1981). While the canonical visual size of an object is related to its real-world size (Konkle & Oliva, 2011), photographs taken so that objects are at their canonical visual sizes will miss an important source of size variability in real-world experience: As people move toward objects, objects change in size while remaining fairly constant in shape and color. We therefore focused on the statistics of the shapes and colors of the salient objects.

### Method

The database was initially created by three individuals at Microsoft, who looked at 200,000 photographs taken from the internet and identified 20,840 photographs which contained a salient object (Liu et al., 2010). Rosenthal et al. (2018) then augmented this database to investigate the color statistics of natural images by having two individuals outline the salient object in each image (i.e., the foreground mask) and assign the salient object a category label. These two tasks were done in separate sessions, with individuals instructed to choose category labels that would allow them to communicate the salient object’s identity to another person.

We filtered the images in this database for category labels tested for in the MCDI to focus on the earliest learned categories. Restricting category labels to those used for at least four images, there were 107 category labels tested for in the MCDI. A total of 7,955 images remained, with a median of 18 images per category. Six images appeared in both a specific category and in a more general category (e.g., “plant” and “flower”), and we retained both labels for each of these six images.

Color was measured for each pixel in the image along three dimensions: lightness, chroma, and hue in the Commission Internationale de l'Eclairage (CIE) lightness-chroma-hue (LCh) color space. The CIE LCh color space is a polar transformation of the CIE Lab color space that aligns with the dimensions commonly used in psychological studies (Gravesen, 2015; Meyer & Greenberg, 1980). However, our values are an approximation as the images are given as device-dependent red-green-blue (RGB) pixel values which can only be mapped to a device-independent color space, such as CIE LCh, with additional information (Wyszecki & Stiles, 1982). For each of the three color dimensions, the mean value across all foreground mask pixels was used as a summary measure.

There are many ways in which shape can be characterized, and in computer vision shape descriptors fall into two main classes: Contour methods that characterize the boundary of the shape and region-based methods that characterize the position of all of the pixels in the shape. While boundary descriptors have been more commonly used in psychological research (e.g., Op de Beeck et al., 2003), they are also less robust to the noise found in natural images than the more general-purpose region-based methods (Zhang & Lu, 2004). We used a region-based approach, Hu moment invariants, as our shape descriptors: A set of seven orthogonal shape dimensions that are independent of object translation, scaling, and rotation (Hu, 1962). Image moments are weighted averages of pixel intensities, with a particularly simple example being the area of the foreground mask, and Hu moment invariants are a set of arithmetic combinations of image moments designed to have the desirable properties listed above. We applied these shape descriptors to the binarized foreground masks.

**Figure 8 fig8:**
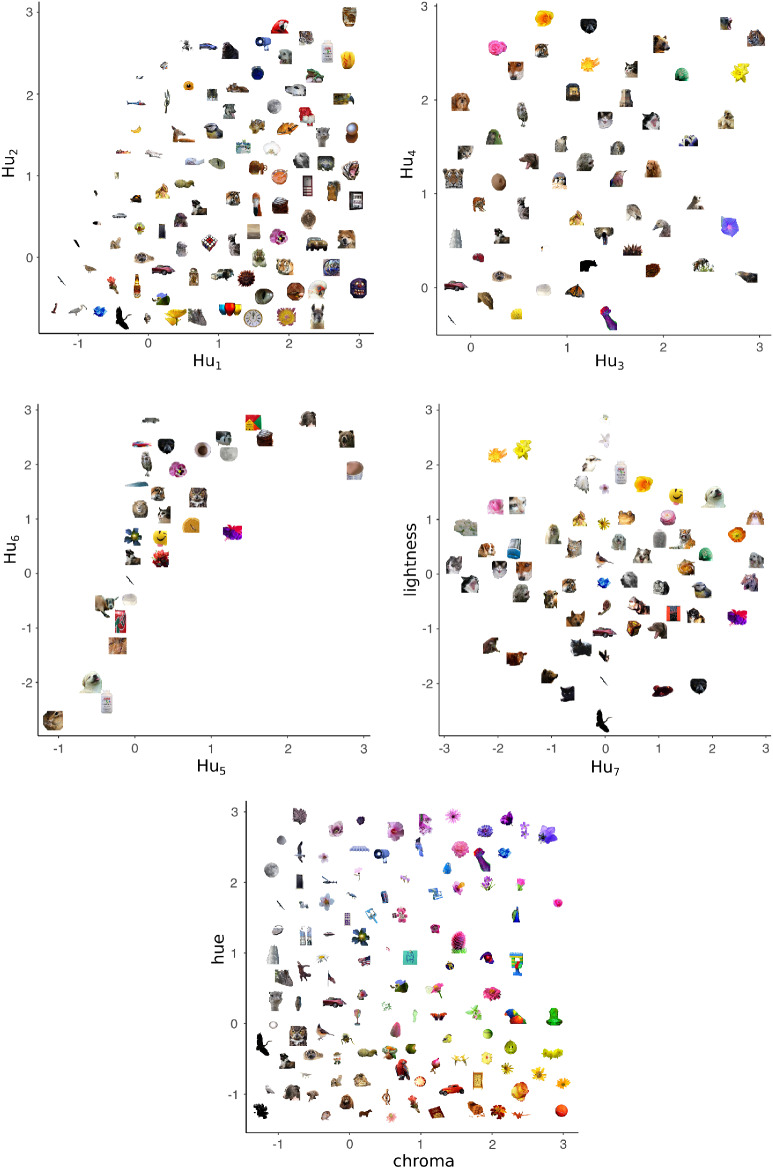
Illustration of the Shape (i.e., The Seven Hu Moment Invariants) and Color Dimensions (i.e., Lightness, Chroma, and Hue) *Note*. Each dimension appears in one of the five panels, and each panel shows how a (normalized) dimension pair differentiates example images. See the online article for the color version of this figure.

Because many of the measurements of color and shape were on incomparable scales, we normalized each of the ten dimensions by subtracting its overall mean and dividing by its overall standard deviation. Figure 8 gives examples of the salient objects plotted along pairs of normalized shape and color dimensions.[Fig-anchor fig8]

### Results and Discussion

**Figure 9 fig9:**
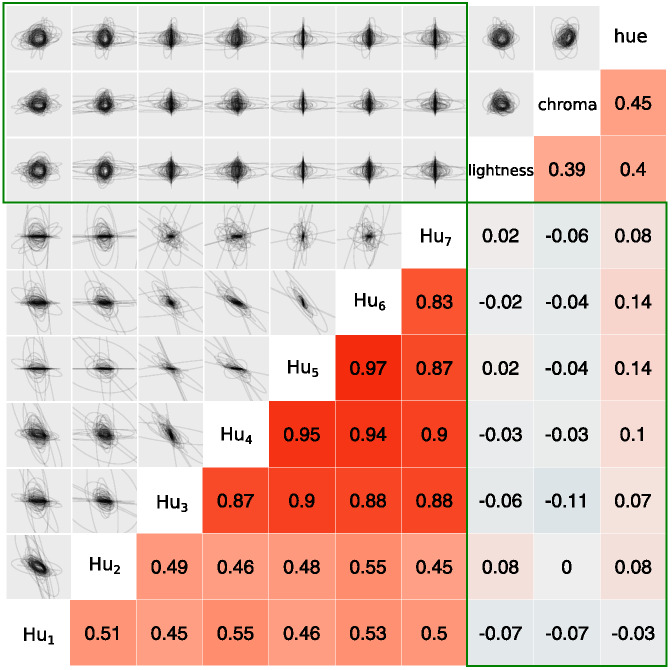
Pairwise Dimension Variability for Categories of Natural Images *Note*. Upper triangle shows equiprobability ellipses for each category for each pair of dimensions. Lower triangle shows Spearman correlations across categories between the standard deviations along each pair of dimensions. Stronger correlations are shaded redder. Green boxes surround the plots of pairs of dimensions usually deemed separable, and these correlations are lower as was hypothesized for separable dimensions. See the online article for the color version of this figure.

We expected that pairs of dimensions usually deemed separable would show covariance structures that were both oriented along the dimensions and also narrow along only one of the dimensions in the pair, as illustrated in Figure 7. We also expected that pairs of dimensions that were deemed integral would show more isotropic covariance structures. Plots that overlay ellipses illustrating each category’s covariance structure are shown for each pair of dimensions in the upper triangle of Figure 9. Shape dimensions paired with color dimensions qualitatively show the anticipated pattern for separable dimensions, while pairs of color dimensions qualitatively show the anticipated pattern for integral dimensions.^4^ We can quantify these observations by correlating the standard deviations along the two dimensions in a pair across all of the categories. In support of Shepard (1987, 1991)’s hypothesis and the assumptions underlying REFRESH, we found positive correlations between pairs of color dimensions and near-zero correlations between color dimensions paired with shape dimensions.[Fig-anchor fig9]

For pairs of shape dimensions, we did not have a strong expectation as to whether they would be separable or integral. Some shape dimensions such as the aspect ratio and curvature of a stimulus are separable, while others, such as radial frequency components, are integral (Op de Beeck et al., 2003). We did not, however, see any identifiable dimensions in Figure 8, suggesting that pairs of Hu moment invariants are perhaps either integral or perhaps are not the dimensions that are used psychologically. The correlations between shape dimension standard deviations in Figure 9 were as high or higher than those for pairs of color dimensions, which by itself suggests integrality. However, a visual inspection of the ellipses in Figure 9 for pairs of shape dimensions seemed to show more “long and thin” ellipses than for pairs of color dimensions, though these long and thin ellipses were not aligned with the axes as they were for color dimensions paired with shape dimensions. This suggests that the psychological dimensions can be approximated as a rotation or other transformation of Hu moment invariant dimensions. While determining the psychological shape dimensions is outside the scope of this work, as a robustness check we performed a principal component analysis of the shape dimensions and reanalyzed the data with these shape dimension principal components. In this reanalysis, we found the same overall pattern of results: Near-zero correlations between shape principal components paired with color dimensions and strong positive correlations between pairs of shape principal components.

### Training REFRESH

To see what REFRESH would learn from natural image statistics, we trained REFRESH on a subset of the dimensions reported in the previous section. We chose the four dimensions of chroma, hue, Hu_3,_ and Hu_4_ as the set of dimensions because Figure 9 suggested that they would illustrate strong pairwise differences. We trained the model on the four physical dimensions and the category labels of 7,955 images, drawing 1,050 samples via Gibbs sampling and discarding the first 50 as burn-in, assuming σ_*r*_^2^ = 1 as above.

We then evaluated what the model had learned by calculating the similarity of new items x* to a single previous item *x* by generalizing the formula for computing similarity using all-or-none consequential regions to graded Gaussian clusters (Shepard, 1987; Tenenbaum & Griffiths, 2001a). Similarity is assumed to be equal to the probability that x* is in the true cluster *C* from which *x* was drawn

$$p(x^{*}\in C|x)=\int_{h\in ℋ}p(x^{*}|x,\;h)p(h|x),$$ 5

where *h* is a cluster and ℋ is the set of clusters under consideration.

**Figure 10 fig10:**
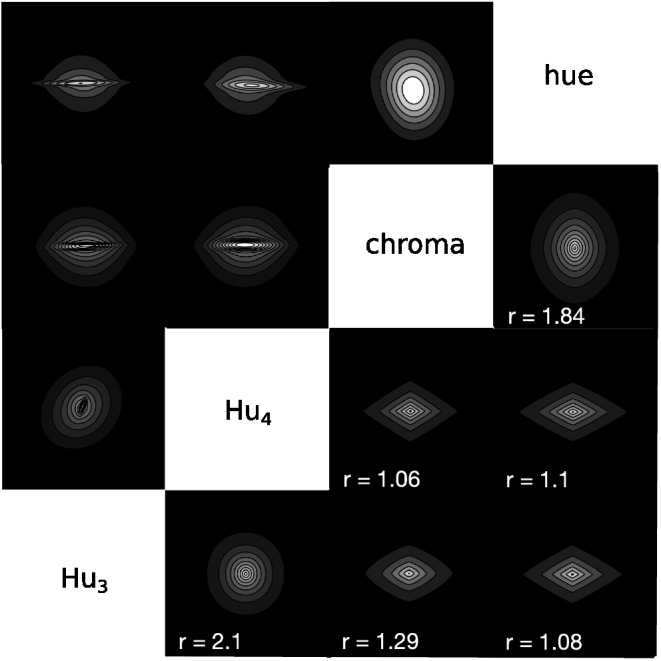
The Effect of Natural Image Statistic Training on the Rational Exclusively Family RESemblance Hierarchy’s Predictions for Between-Example Similarity *Note*. Each plot in the upper triangle shows REFRESH’s resulting similarities between a new example and an example in the center of the plot. Examples vary along two dimensions with the remaining dimensions held constant at their mean values. In the contour plots, lighter colors represent higher similarities, and the lines are iso-similarity curves. Each plot in the lower triangle shows the best-fitting Minkowski metric for the similarities in the corresponding plot in the upper triangle, with the best-fitting exponent *r* printed in the corner of the plot.

The iso-similarity curves that REFRESH produces after training are shown in the upper triangle of Figure 10. The curves best resemble the Euclidean metric for pairs of color dimensions and for pairs of shape dimensions, but appear non-Euclidean for color dimensions paired with shape dimensions. We fit a Minkowski distance metric to each iso-similarity plot^5^ and display the iso-similarity curves of the best-fitting metric in the plots in the lower triangle along with the exponent *r* printed in the corner of each plot. The recovered exponents were close to 2 for pairs of color and pairs of shape dimensions, but close to 1 for color dimensions paired with shape dimensions.[Fig-anchor fig10]

Importantly the different metrics are present in the model simultaneously: Each iso-similarity plot depicts a two-dimensional “slice” of the entire four-dimensional iso-similarity space with the remaining variables fixed at their mean values. This provides an interesting illustration of how REFRESH can match empirically observed similarity metrics by training on natural image statistics, though further work will be needed to establish the extent to which these results are robust to changes in model parameters and the specific image statistics chosen.

We next investigated what REFRESH would learn from artificial categories, which allowed us to carefully control the statistics of the stimuli within a two-dimensional stimulus space. As a caricature of the variability of natural categories, we assume that there are two kinds of clusters within this space: One kind that has low variability on the vertical dimension but with high variability on the horizontal, while the other category type has low variability on the horizontal dimension but high variability on the vertical. For the simulations below, we assumed that for each cluster 30 training data points were drawn from a Gaussian distribution.

**Figure 11 fig11:**
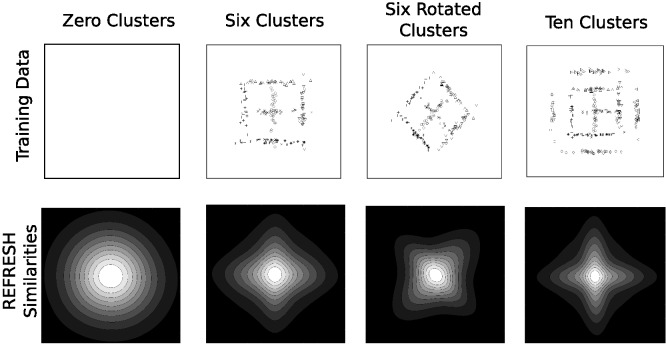
Illustration of Training Examples and Resulting Predicted Similarities Between a New Example and an Example in the Center of the Plot *Note*. Different marker shapes in the training data mark different training contexts, one for each category. In the contour plots, lighter colors represent higher similarities and the lines are iso-similarity curves.

The first column of Figure 11 demonstrates the iso-similarity curves that the model has before training. For this set of simulations, we assumed that σ_*r*_^2^ = 1. We drew 1,050 samples from the model via Gibbs sampling and discarded the first 50 samples as burn-in. The remaining samples were then used to construct the similarity between an item in the center of the plot and an item at that position in the plot. Because the prior over covariance matrices is initially isotropic, before training the iso-similarity curves are circular, reflecting no alignment with the separable dimensions of the space.[Fig-anchor fig11]

However, once the model has been trained on categories that align with the separable dimensions of the space, it then does show an alignment with these separable dimensions. The second column of Figure 11 demonstrates how the iso-similarity curves change when the model has been exposed to data that are drawn from six clusters, three with a standard deviation on the horizontal dimension ten times their standard deviation on the vertical dimension, and vice versa for the other three. Gibbs sampling was initialized with each training point having its own cluster and own component, and each cluster was given a different context. After exposure to these training data, the iso-similarity curves now align with the separable dimensions of the space, with the city-block-like iso-similarity curves reflecting an averaging over the two kinds of variability experienced in the training clusters. This kind of averaging explanation has been advanced by other researchers as a way to produce iso-similarity curves with comparable properties, but these accounts assumed that the separable dimensions were prespecified (Nosofsky, 1986; Shepard, 1987). In contrast, REFRESH’s learned alignment depends entirely on the variability of the clusters: The third column of Figure 11 shows how these iso-similarity curves will be rotated 45 degrees if the training data were generated from Gaussian distributions that are also rotated 45 degrees. The iso-similarity curves now appear to be a city-block-like metric that has been rotated 45 degrees. In addition, the strength of the alignment depends on the number of training clusters. In the fourth column of Figure 11, the model has been trained on ten clusters instead of six. The resulting concavity of the iso-similarity curves is now greater and they appear to be nonmetric (see Figure 5).

The model trained on natural or artificial stimuli is very slow and memory-intensive to simulate, so for many of the simulations below we used an approximation to the trained REFRESH that made the model tractable in sequential tasks. First, we used a particle filter (see Appendix), we assumed ω was equal to the mean stimulus, and we assumed that the covariance of the prior for the cluster means was σ_*r*_^2^*I*, where σ_*r*_^2^ = 0.1. We approximated asymptotic learning of the component covariance distributions by assuming that each IW prior distribution over covariances associated with each component had collapsed to a single covariance matrix. For simplicity, we assumed equal prior probability for each of the components.

We used a standard set of possible covariance matrices across simulations (see Table A1 in the Appendix for the parameterized covariance matrices). These were inspired by the regularities in natural image statistics discussed above, but were specifically chosen to match human behavior across the range of tasks we simulated. For two-dimensional stimuli, we assumed different learned covariance matrices depending on whether the pair of stimuli were separable or integral. The notation we use for all of our parameterized covariance matrices follows Ψ_*x*_, where *x* is replaced by a series of letters referring to the size of the variances along Dimension 1, then Dimension 2, and so forth. Specifically, we used *w* to refer to a dimension with wide variability, and *n* to refer to a dimension with narrow variability. For two-dimensional integral stimuli, we assumed that the model had been trained on categories that were isotropic, so for stimuli using a pair of integral dimensions, we assumed that only Ψ_*ww*_ is available: The variances are wide along both dimensions. For two-dimensional separable stimuli, we assumed that there were two possible covariance matrices available: Ψ_*nw*_ and Ψ_*wn*_. These reflect the same environmental regularities we used in the training stimuli above, where categories were assumed to be aligned with the separable dimensions of the space and *singly narrow*: One was narrow along one dimension and the other narrow along with the other. For both Ψ_*nw*_ and Ψ_*wn*_, we assumed that the standard deviation along the narrow dimension was equal to 10% of the size of the standard deviation along the wide dimension. Even with these approximations, it is impractical to use a fitting algorithm to match the empirical data, so we attempted to best match the data by adjusting by hand the scale parameters that each standard deviation is divided by, *c*_*d*_, associated with each dimension *d* (see Table A1 in the Appendix). Larger values of the scale parameters *c*_*d*_ generally result in smaller clusters and in REFRESH learning categories more quickly. Except where noted, we assumed that these parameters were equal across dimensions.

For tasks with three-dimensional stimuli, we assumed a larger set of possible three-dimensional covariance matrices. For integral stimuli, we assumed that the model had been trained on categories that were isotropic and for stimuli using a pair of integral dimensions we assumed that only Ψ_*www*_ is available. For separable dimensions we generally assumed that there were a set of singly narrow components available for each dimension, reflecting training with categories for which only one of the three dimensions is near-constant. For these singly narrow covariance matrices describing such categories, Ψ_*nww*_, Ψ_*wnw*_, and Ψ_*wwn*_, we assumed that the two larger standard deviations were equal, and that the single smaller standard deviation was 10% of the size of two larger standard deviations. For the three-dimensional stimuli, we also include a set of *doubly narrow* covariances. For these doubly narrow components, Ψ_*wnn*_, Ψ_*nwn*_, and Ψ_*nnw*_, we assumed that the two smaller standard deviations were equal to 30% of the larger standard deviation. We found it was necessary to use 30% instead of 10%, otherwise the doubly narrow components tend to dominate the singly narrow components, as the probability of a stimulus located at the center of a cluster is much higher.^6^ It is possible that this reflects a natural regularity, though we did not find any evidence in our analysis of natural images statistics that category structures follow this pattern. Alternatively, it is possible that these parameters have an algorithmic-level justification reflecting attentional capacity limits. As discussed above, the covariance matrices act to stretch or shrink the space of stimuli, as selective attention does. Selective attention is assumed to have a capacity limit that imposes trade-offs between dimensions, and having the smaller standard deviation be larger for doubly narrow covariance matrices than for singly narrow covariance matrices also corresponds to a capacity limit.^7^ As with the two-dimensional matrices, we introduced a set of *c*_*d*_ parameters in each simulation to scale the matrices along each dimension.

We ensured consistency between the two-dimensional and three-dimensional covariance matrices, as we assumed that they result from exposure to the same real-world categories. The projections of the first two dimensions of Ψ_*www*_, Ψ_*nww*_, or Ψ_*wnw*_ are equivalent to Ψ_*ww*_, Ψ_*nw*_, and Ψ_*wn*_, respectively. In the two-dimensional simulations, however, we did not include any projections of Ψ_*wnn*_, Ψ_*nwn*_, or Ψ_*nnw*_ as these are not as narrow along their narrowest dimension, so considering only projections of a narrow with a wide dimension, the model is much more likely to choose Ψ_*www*_, Ψ_*nww*_, or Ψ_*wnw*_ instead to describe clusters of stimuli. Ensuring consistency is the most compelling reason for setting the narrow variances in the doubly narrow components to be wider than the narrow variance in the singly narrow components: otherwise the projection of the two narrow dimensions from a doubly narrow component would dominate the singly narrow components for two-dimensional stimuli, and this dominant isotropic projection would not allow the model to explain dimensional biases.

## Explaining Dimensional Biases Using REFRESH

Having developed REFRESH, we now use this model to explain the classic dimensional biases, how the dimensional biases are stimulus dependent, and how the dimensional biases can be learned.

### Explaining the Classic Dimensional Biases

#### Violations of the Triangle Inequality

Before training, REFRESH will not violate the triangle inequality. The iso-similarity curves in the untrained REFRESH are circular (see first column of Figure 11) and the circular iso-similarity curves will not violate the triangle inequality (see Figure 5). With training, with two types of variability we assumed above, REFRESH can potentially violate the triangle inequality, but it depends on the quality and quantity of the training that is given. The trained REFRESH iso-similarity curves are essentially the average of the priors learned for the clusters with the two different kinds of variability in the training data. One type of prior expects high variance along the vertical dimension and low variance along the horizontal dimension, while the other type expects high variance along the horizontal dimension and low variance along the vertical dimension. With categories showing the variability of the six clusters example in Figure 11, averaging the prior components produces an iso-similarity metric that appears city-block, and so the triangle inequality would not be violated. However, when trained on additional categories as in the 10 clusters example in Figure 11, averaging the prior components produces an iso-similarity metric that appears nonmetric, thus violating the triangle inequality (see Figure 5).

Empirically, there are dimensions for which similarity judgments violate the triangle inequality to a greater or lesser extent (Tversky & Gati, 1982). The perceptual dimensions that showed the strongest violations of the triangle inequality were those for which different parts of the stimuli changed shape, while the evidence for violations of the triangle inequality for squares that varied in size and brightness was mixed, and no violations of the triangle inequality were observed for squares that varied in hue and chroma. REFRESH predicts that violations of the triangle inequality should depend on the number of categories aligned with a single dimension (see Figure 11).

Interestingly, the trained REFRESH will also produce different types of iso-similarity curves depending on whether the stimuli are easily distinguishable or not. The iso-similarity curves produced in the 10 clusters example of Figure 11 are concave for stimuli far from the central stimulus and convex for stimuli that are close to the central stimulus, matching the empirical pattern found across studies (Nosofsky, 1986; Tversky & Gati, 1982).

#### Condensation Versus Filtration

**Figure 12 fig12:**
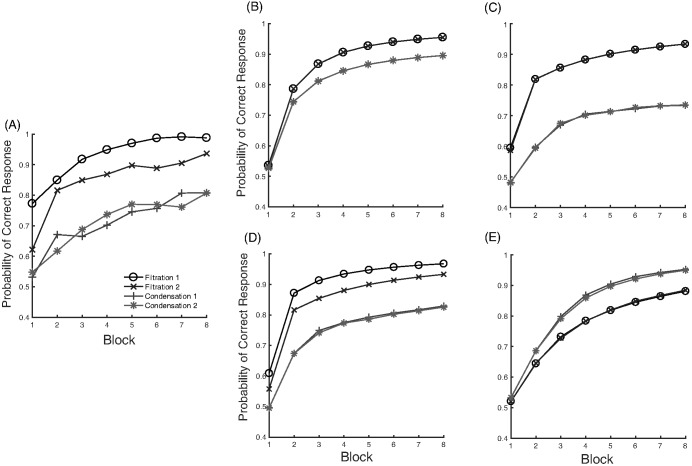
Human Data and Model Results for the Condensation Versus Filtration Task (See Figure 2) *Note*. Each block consists of all eight stimuli presented in a random order. (A) Human data from Kruschke (1993), adapted with permission from page 15. (B) Predictions from the Rational Model of Categorization, which match the human pattern for condensation versus filtration but show no difference between dimensions. (C) Predictions from REFRESH assuming covariance components narrow along one or the other dimension, which match the human pattern for condensation versus filtration but show no difference between dimensions. (D) Predictions from REFRESH assuming covariance components narrow along one or the other dimension, but with narrower covariances on the vertical dimension. This result matches both the human pattern between condensation versus filtration and also the difference between dimensions. (E) Predictions from REFRESH assuming an isotropic covariance component, which shows the reverse pattern from the human data.

Now we turn to the next classic dimensional bias: condensation versus filtration. The human data show an advantage for the filtration categories (see Figure 12A), and as discussed above, the RMC does produce the empirical ordering of the conditions (see Figure 12B). For REFRESH, we assume that the effect of training with two types of dimensionally aligned categories, as we assumed for violations of the triangle inequality, results in two components: Ψ_*nw*_ and Ψ_*wn*_ (see Table A1 in the Appendix for details). Using *c*_1_ = *c*_2_ = 0.5 to approximately match the overall level of human accuracy, REFRESH finds the filtration categories easier to learn than the condensation categories (see Figure 12C). This is because Ψ_*nw*_ and Ψ_*wn*_ are helpful if the decision boundary is parallel to one of the dimensions but are detrimental if the decision boundary cuts across the dimensions. An even better match of REFRESH to human data can be found if we assume greater discriminability along the vertical dimension, with *c*_1_ = 0.5 and *c*_2_ = 1 (see Figure 12D). More broadly, we would expect that REFRESH would also reproduce the qualitative changes shown in categorizing transfer stimuli that empirically depend on whether the category boundaries are aligned with the separable dimensions of the space (McKinley & Nosofsky, 1996).[Fig-anchor fig12]

#### SHJ Type II Advantage

**Figure 13 fig13:**
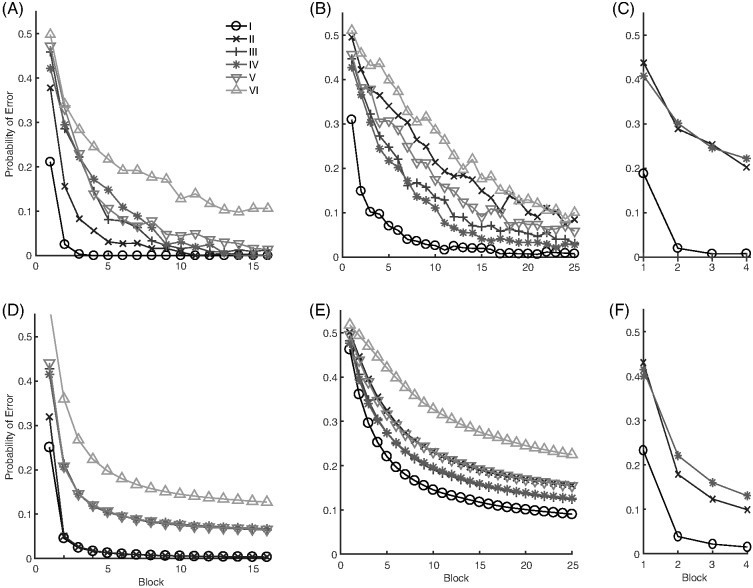
Human Data (Top Row) and Model Fits (Bottom Row) to Errors in Three Different Versions of the Shepard et al. (1961) Task *Note*. Each block consists of 16 stimuli (i.e., two copies of the eight possible stimuli) in a restricted random order. (A) Human data from an experiment using separable dimensions, adapted with permission from page 355 of Nosofsky, Gluck, et al. (1994). (B) Human data from an experiment using integral dimensions, adapted with permission from page 225 of Nosofsky and Palmeri (1996). (C) Human data from an experiment using separable dimensions in which there was no Type II advantage, adapted with permission from page 564 (Experiment 6) of Kurtz et al. (2012). (D) REFRESH results assuming both singly narrow and doubly narrow covariance components. (E) REFRESH results assuming only isotropic covariance components. (F) REFRESH results assuming singly narrow, but not doubly narrow covariance components.

**Figure 14 fig14:**
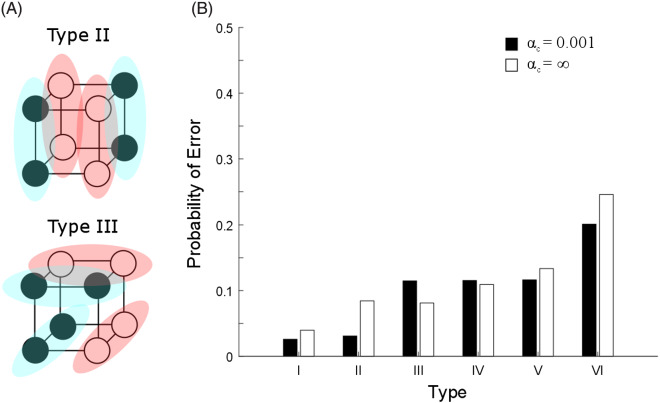
Clustering Illustration for SHJ Problems and Model Predictions When the Contextual Prior Is Removed *Note*. (A) Illustration of the clustering of Types II and III. Each colored blob is a cluster with different colors associated with different categories. (B) Model predictions when the contextual prior is present (α_*c*_ = 0.001) or removed (α_*c*_ = ∞), with errors averaged over the first 16 blocks. See the online article for the color version of this figure.

In experiments with the SHJ types, the classic result is that errors are lowest for Type I and generally increase across types: Type I < Type II < Types III–V < Type VI (Nosofsky, Gluck, et al., 1994; Shepard et al., 1961; see Figure 13A). The classic dimensional bias is shown by the advantage that Type II has over Types III–V, because based on family resemblance (e.g., pairwise stimulus confusability) alone it should be worse. Exemplar models produce the SHJ Type II advantage using selective attention to the separable dimensions—learning Type II requires attending to only two of the three stimulus dimensions, so the model devotes more attentional resources to the relevant dimensions. The trained REFRESH produces the Type II advantage because its set of covariance matrices embody similar functional limits on how much a set of dimensions can be stretched or shrunk. For these three-dimensional SHJ stimuli we used the trained three-dimensional covariance matrices for separable stimuli: the singly narrow components Ψ_*nww*_, Ψ_*wnw*_, and Ψ_*wwn*_, as well as the doubly narrow components Ψ_*wnn*_, Ψ_*nwn*_, and Ψ_*nnw*_. Looking at the structure of the problems in Figure 3, intuitively the singly narrow components will be very useful for speeding learning of Type I problems but will not be particularly helpful for Type II problems. The Type II advantage instead depends on the doubly narrow components, as these covariance matrices can describe the Type II category well as shown in Figure 14A. Using *c*_1_ = *c*_2_ = *c*_3_ = 2.1 to match the speed of human learning leads to REFRESH producing a very big Type II advantage (see Figure 13D), in contrast to the RMC which can only produce a limited Type II advantage (Nosofsky, Gluck, et al., 1994). This Type II advantage is actually somewhat too large as there is now little advantage of Type I over Type II. However, the performance of Type II can be made to fall between that of Type I and Types III–V by increasing the relative width of the doubly narrow components to a larger value (e.g., 60% instead of 30% of the largest standard deviation).[Fig-anchor fig13] [Fig-anchor fig14]

The influence of REFRESH’s hierarchical prior on components helps explain the mechanism used to produce the SHJ Type II advantage. This part of the prior biases the model to use only one or a few components in each experimental context. This prior could potentially reflect the structure of the natural environment, though this is entirely speculative as our natural image analysis does not speak to this. Alternatively, it could reflect a limit of cognitive capacity. As can be seen in Figure 14B, when this contextual prior is removed (which can be done by setting α_*c*_ = ∞ and keeping the other parameters constant), then Type III performance is as good or better than the Type II performance. This is for the same reason that Boolean complexity evaluates Type II and Type III as equally difficult: Both can be perfectly captured by two doubly narrow clusters within each category (Feldman, 2000; Vigo, 2006), as shown in Figure 14A. The difference between Type II and III is that the clusters needed for Type II are all from the same component, while two different components are needed for Type III. The usual contextual prior distribution (α_*c*_ = 0.001) penalizes using multiple components, a mechanism that has been used by some rule-based models to explain the advantage of Type II over Type III (Goodman et al., 2008). REFRESH produces the SHJ Type II advantage for the same reason as rule-based and selective attentional models: that there is either a bias to using a single kind of rule, or there is a single way in which selective attention stretches or shrinks all of the stimuli. Therefore, it is important that α_*c*_ be set to a low value for REFRESH to reproduce the human ordering of SHJ types.

### Explaining the Stimulus-Dependence of Dimensional Biases

#### Separable Versus Integral Dimensions

**Figure 15 fig15:**
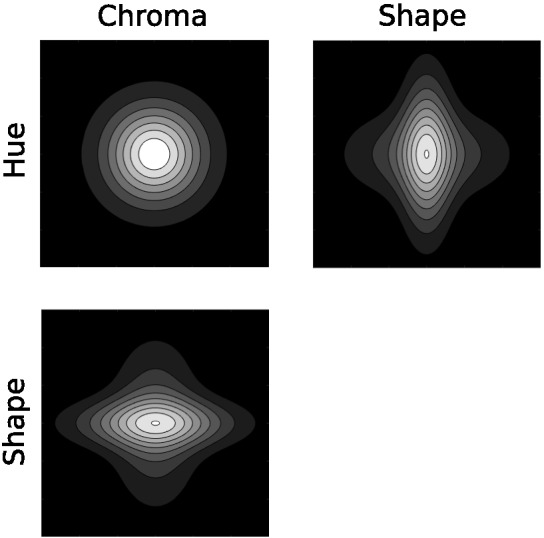
Illustration of how the Relationships Between Three Variables, Hue, Saturation, and Size, Can Be Both Separable and Integral in the Rational Exclusively Family RESemblance Hierarchy *Note*. Similarities are between a new example and an example already in the center of the plot. In the contour plots, lighter colors represent higher similarities, and the lines are iso-similarity curves. Each plot shows a pair of dimensions.

As discussed above, a pair of dimensions such as hue and chroma are integral, but if either hue or chroma are paired with a shape dimension then the resulting pair are separable dimensions. The trained REFRESH can capture these different pairwise relationships. For example, assume that the stimulus dimensions are hue, chroma, and shape in that order and that the experienced categories only followed a subset of the three-dimensional covariances used above: Ψ_*wwn*_ and Ψ_*nnw*_. Figure 15 shows the pairwise iso-similarity curves that are predicted by REFRESH: They resemble the Euclidean metric for hue paired with chroma at all levels of similarity, but resemble different metrics depending on the level of similarity for both hue paired with shape and chroma paired with shape: Euclidean for high similarity, city-block for intermediate similarity, and an nonmetric for low similarity. This result is due to Ψ_*wwn*_, and Ψ_*nnw*_ both being isotropic when restricted to the first two dimensions, but being similar in structure to the pair Ψ_*wn*_ and Ψ_*nw*_ when looking at either the first or second dimension paired with the third dimension. Indeed, these plots resemble those of Figure 10 for the separable dimensions in which it appears that there are mixtures of two components: a narrower component along the shape dimensions than along the hue or chroma dimensions.[Fig-anchor fig15]

Both the condensation versus filtration effect and the SHJ Type II advantage depend on whether the dimensions used are separable or integral. Empirically, the advantage of filtration categories over condensation categories is reversed for integral dimensions (Gottwald & Garner, 1975). To match this result with REFRESH, we assume that only the isotropic trained component covariance, Ψ_*ww*_, is available and that *c*_1_ = *c*_2_ = 2. As a result, REFRESH also produces an advantage for the condensation categories over the filtration categories (see Figure 12E). Likewise, for the SHJ problems, when using integral dimensions such as the hue, saturation, and brightness of color chips, the empirical effect changes (Nosofsky & Palmeri, 1996): Type II problems become relatively more difficult to learn (see Figure 13B). This dependence of the Type II and Type IV ordering has been explained by assuming that selective attention is easier with separable than integral dimensions (Nosofsky & Palmeri, 1996), and REFRESH produces this effect for an analogous reason: that there is a single isotropic covariance component, Ψ_*www*_ (with *c*_1_ = *c*_2_ = *c*_3_ = 2.5), so it is not possible to utilize a more advantageous component (see Figure 13E).

Note that REFRESH makes a distinction between novel dimensions and those that have been trained to be integral. We argue that while people have experienced many categories with stimuli that vary along the common integral dimensions such as hue, saturation, and brightness, these categories do not have the strong differentiation in types of variability seen for separable dimensions. Instead, we assume that there is a common type of variability that would result in REFRESH having a strong isotropic prior, like Ψ_*www*_, an assumption which is supported by the isotropic category statistics we found for color dimensions in Figure 9. Of course, we should note that our assumption of an isotropic covariance for color is only a rough approximation, as researchers have found indications of preferred dimensions in the color space, though not to the extent that they can be called separable (Burns & Shepp, 1988; Ell et al., 2012; Foard & Kemler Nelson, 1984; Nosofsky, 1987). It may be that the color space is better described by the same kinds of covariance matrices that are used for separable dimensions, but with the narrow variances being less narrow so that each is more isotropic. Indeed, from the point of view of REFRESH, the distinction between integral and separable dimensions is not absolute, but rather a matter of degree.

**Figure 16 fig16:**
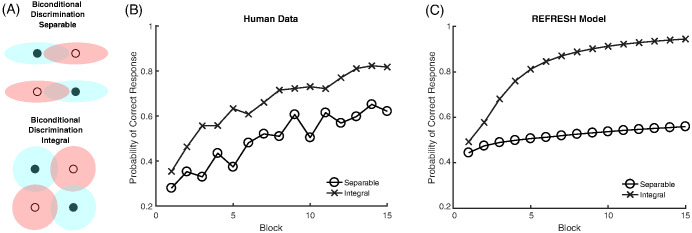
Human Data and Rational Exclusively Family RESemblance Hierarchy Results for the Biconditional Discrimination Experiment With Both Separable and Integral Stimuli of Soto et al. (2015) *Note*. (A) Illustration of the stimuli parameters with circle color indicating category label. REFRESH’s cluster expectations (blue and pink shades) differ for separable and integral stimuli. (B) Data from Soto et al. (2015) Experiment 2, which consisted of 15 training blocks with all four stimuli presented in each block. Adapted with permission from page 172. (C) REFRESH results for this same experiment. See the online article for the color version of this figure.

In contrast to a strong isotropic prior, Soto et al. (2015) argued that the *direction hypothesis* explained results with integral dimensions: that for integral dimensions there are effectively a very large number of available components that are aligned in all possible dimensions. Using a clever experimental design that ensured that the psychological distances between stimuli were equivalent for integral and separable stimuli, they showed that biconditional learning was faster for integral stimuli (see Figure 16B). However, the authors noted that exemplar models could potentially explain these results as a consequence of using a city-block metric for separable dimensions and a Euclidean metric for integral dimensions, though they criticized this explanation as being a redescription of the generalization gradients. As REFRESH produces these generalization gradient differences as a result of training across categories, it also can produce the integral dimension advantage for biconditional discrimination (see Figure 16C). For this simulation, we assumed that, as in the discussion of the pairwise relationship between separable and integral dimensions above, the two available components were Ψ_*wwn*_ and Ψ_*nnw*_ and also that *c*_1_ = *c*_2_ = *c*_3_ = 1.3. To describe the combination of a separable dimension with an integral dimension, the first dimension was paired with the third dimension (which is equivalent to the second dimension paired with the third dimension), while to describe the combination of two integral dimensions, the first two dimensions were paired. The intuition for this result is that the trained components are aligned with the separable dimensions and thus are more likely to overlap with stimuli from the other category than the isotropic components used for integral dimensions are (see Figure 16A). Compared to integral dimensions, the higher overlap for separable dimensions reduces performance. This provides an alternative explanation for the results of Soto et al. (2015) that is similar to that of an exemplar model but ascribes the difference in generalization gradients to differences in past training.[Fig-anchor fig16]

#### SHJ Type II Advantage Dependencies

While SHJ performance depends on whether dimensions are separable or integral, it also depends on how the Type II category structure is mapped to the stimulus dimensions. This can be done in one of three ways, resulting in either the size, shape, or color dimensions being irrelevant to learning the categories. Comparing these different mappings, Kurtz et al. (2012) showed in three experiments that only the size-irrelevant subtype showed a Type II advantage, while the other types did not. However, in contrast, using the same dimensions, Love and Markman (2003) found in two separate experiments that the shape-irrelevant subtype showed a Type II advantage, while the other types did not. This mix of consistency and variability across studies suggests that behavior is influenced by some subtle aspect of the stimuli or task, and indeed the work of Mathy and Bradmetz (2011) supports this: finding that the Type II advantage depends on the materials and the spacing between stimuli. They replicated the shape-irrelevant subtype advantage of Love and Markman (2003) for some materials, but showed that the shape-irrelevant subtype performance depended on how similar the two values of shape were: the closer the shapes were to one another, the better the shape-irrelevant subtype performed. The effect of increased similarity on the irrelevant dimension resulting in better Type II performance was later replicated (Mathy et al., 2013).

**Figure 17 fig17:**
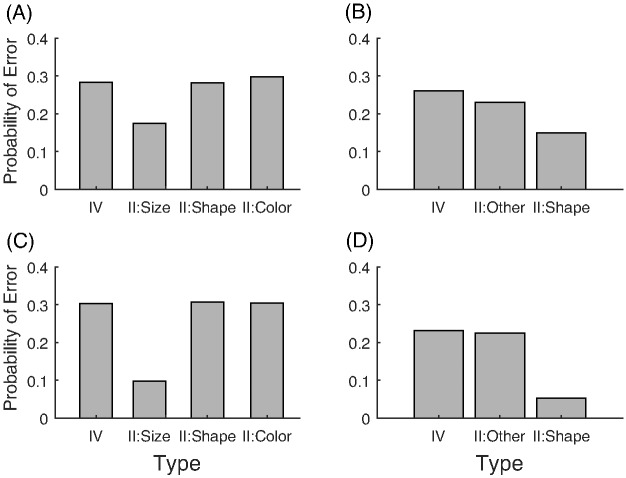
Human Data and Rational Exclusively Family RESemblance Hierarchy Results for how the SHJ Type II Advantage Depends on the Mapping and Distance Between Stimuli *Note*. (A) Data from Kurtz et al. (2012) averaged over Experiments 1, 2, and 4, which were each 64 trials long. (B) Data from Love and Markman (2003) Experiment 1, which was 128 trials long. (C) REFRESH results for the first 64 trials when stimuli are closer to the size dimension. (D) REFRESH results for the first 128 trials when stimuli are closer to the shape dimension.

The result that performance increases as values along the irrelevant dimension become more similar goes against the predictions of the basic exemplar model. But this finding, along with the finding that separating out the relevant dimensions improves Type II performance, arises naturally from REFRESH. As can be seen in Figure 14A, doubly narrow components will only capture pairs of stimuli if they are spaced closely enough on the irrelevant dimension. This in turn can explain why only one Type II subtype shows an advantage over Type IV: On one of the dimensions the stimuli are closer than on the other two. To model this, we assumed REFRESH could use all the singly narrow and doubly narrow covariances, that the dimensions were size, shape, and color in that order, and that *c*_1_ = 0.7 while *c*_2_ = *c*_3_ = 2.3. Because the distance is less on the size dimension, then there is an advantage for the size-irrelevant subtype (see Figure 17C). To produce an advantage for the shape-irrelevant subtype, we used all the same parameters except that *c*_2_ = 0.7 while *c*_1_ = *c*_3_ = 2.3 (see Figure 17D).[Fig-anchor fig17]

The SHJ Type II advantage also depends on the set of separable dimensions used. In their Experiment 6, Kurtz et al. (2012) used the dimensions of border color (yellow or white), interior dots (present or absent), and interior diagonal line (present or absent), rather than the usual dimensions of size, shape, and color. The results were a surprising combination of what has been seen with separable and integral dimensions: Type I performance was excellent as would be expected for separable dimensions, but there was no Type II advantage over Type IV (see Figure 13C). When fitting ALCOVE to these data, Kurtz et al. (2012) found that it could capture either the fast learning of Type I compared to Types II and IV, or the tie between Types II and IV, but not both.

To explain these results, we appeal to intuitions about natural-category statistics. While above we have proposed both singly narrow and doubly narrow components for the dimensions of size, shape, and color, the categories experienced along the dimensions of border color, interior dots, and interior diagonal line are probably different. We can think of examples in which we have experienced singly narrow categories along these dimensions. For example, border color can be consistent across a set of web pages on the same site, interior dots could be consistent among those with a disease, and the presence or absence of a diagonal line on a uniform can define different kinds of military personnel. However, compared with size, shape, and color, it is much harder to think of doubly narrow categories. Experience with singly narrow categories will produce separable dimensions, and if we use only the singly narrow covariances, Ψ_*nww*_, Ψ_*wnw*_, and Ψ_*wwn*_, as well as *c*_1_ = *c*_2_ = *c*_3_ = 2.5, then REFRESH produces both relatively fast learning of Type I as well as a much reduced Type II advantage (see Figure 13F).

### Explaining how Dimensional Biases Can Be Learned

All of the above demonstrations of REFRESH producing the classic dimensional biases and their stimulus dependence are the result of learning. Rather than being fixed properties of the categorization system, these results are, we suggest, dependent on the cognitive system learning the statistical structure of real-world categories. Here we first focus on two key effects that we reviewed earlier showing the developmental trajectory of these biases: dimensional learning and the differential development of SHJ Type I and II. Next, we focus on how categorization training changes dimensional biases, both for dimensions considered integral and also for novel dimensions defined arbitrarily.

#### Dimensional Development

**Figure 18 fig18:**
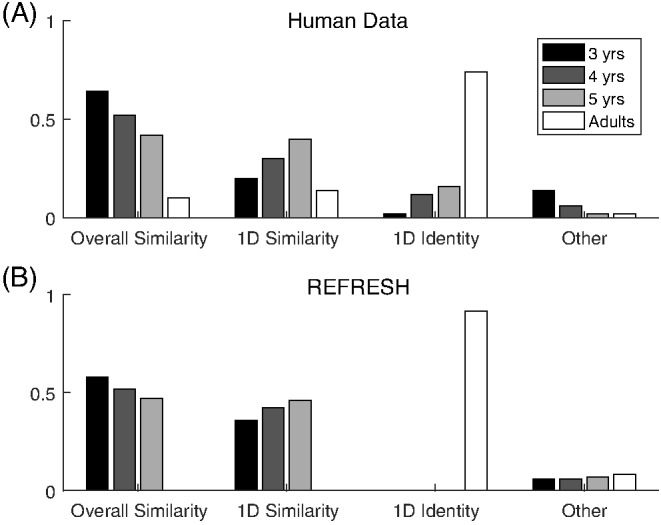
The Developmental Trends of Dimensional Learning From Experiment 2 of Smith (1989) *Note*. Bar plots display proportions of each group producing the critical partitions shown in Figure 4, where bar color indicates the age group. Both the (A) human data (adapted with permission from page 138) and (B) REFRESH results show a trend over developmental time of moving from clustering objects according to overall similarity to clustering them by matching a single feature.

Over the course of development, there are changes in how people decide that two stimuli are the same. Initially distance within the psychological space is most important, but gradually exact matches along separable dimensions take precedence. Smith (1989) clearly demonstrated this developmental trajectory using the stimuli shown in Figure 4. Participants in this free classification task tended to group the six stimuli in three different ways: overall similarity, one-dimensional similarity, and one-dimensional identity. Three- and 4-year olds were most likely to use overall similarity, 5-year olds were almost as likely to use one-dimensional similarity as overall similarity, and adults overwhelmingly used one-dimensional identity (see Figure 18A). This experiment was a particularly nice demonstration of the developmental trend because the overall similarity results in this experiment required using both dimensions. Similar claims made with simpler category structures (e.g., Smith & Kemler Nelson, 1984) have been criticized because results attributed to overall similarity can also be produced by a focus on a single dimension (Thompson, 1994; Wills et al., 2015).[Fig-anchor fig18]

To see how this result arises from REFRESH, we first note the earlier demonstration of how the covariance components change with experience with categories that are narrow along a single dimension (see Figure 11): they begin with an expectation that categories are isotropic, and the variance of the component will gradually narrow in response to more and more training. We assumed that adults used Ψ_*nw*_ and Ψ_*wn*_, and also assumed that the other age groups had less experience with categories and so their variances along the narrow dimension for Ψ_*nw*_, and Ψ_*wn*_ were not quite as narrow. In particular, we assumed variance decreased along the narrow dimension as children grew older and gained more experience: 0.8 for 3-year olds, 0.6 for 4-year olds, 0.5 for 5-year olds, which were all wider than the 0.1 value used for adults.^8^ For simplicity, for all age groups we assumed that the two dimensions were equally scaled: *c*_1_ = *c*_2_ = 2.3.

REFRESH automatically infers how the stimuli are clustered, so we merely report the probability of different partitions of the stimuli into clusters as the results of the simulation. For this simulation, we were able to exactly calculate the probability of each of the partitions because there were only six stimuli in the experiment (see Appendix). However, as almost all of the different ways of partitioning the stimuli would be classified as “other” and only a small number would be classified as either overall similarity, one-dimensional similarity, or one-dimensional identity, we had to use slightly different parameters from the previous simulations to ensure that REFRESH produced these interesting partitions and not near misses. For this simulation we changed the chance of creating new clusters and the variance of the positions of the components: α = 1, σ_*r*_^2^ = 100. Using these values, REFRESH shows a smooth transition from using overall similarity to cluster the items, to one-dimensional similarity, and then finally to one-dimensional identity for adults (see Figure 18B).

The developmental data for the SHJ problems show a similar picture to the developmental data for free classification. Experiment 1 of Minda et al. (2008) investigated SHJ problems with 3-year olds, 5-year olds, 8-year olds, and adults. For Type I problems, all of the groups were able to learn the categories fairly quickly, except for the 3-year olds. This result is congruent with the highest proportion of overall similarity partitions occurring for 3-year olds in the Smith (1989) data. Interestingly, though 5-year olds and 8-year olds were much better on Type I problems compared to 3-year olds, they were similar to 3-year olds in their Type II performance, where adults performed better. All age groups produced similar accuracy on Type IV problems, suggesting that motivation and understanding of the instructions were similar across groups.

**Figure 19 fig19:**
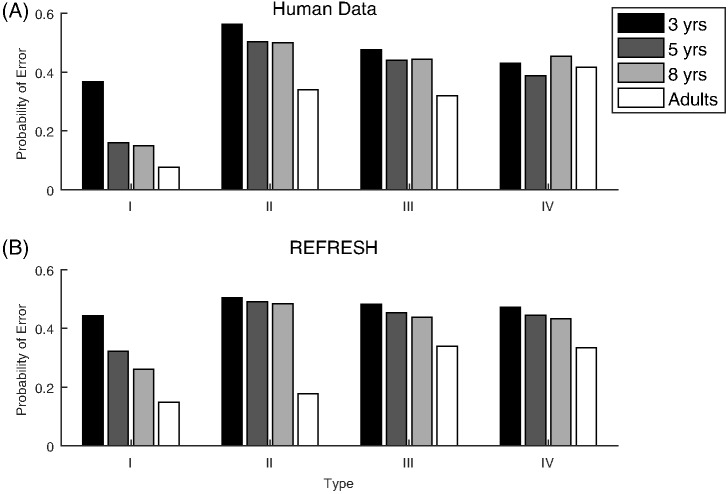
The Developmental Trends of Errors for the SHJ Problem Types From Experiment 1 of Minda et al. (2008) *Note*. Bar plots display the average error for SHJ Types I–IV over 48 training trials on each type for 3-year olds, 5-year olds, 8-year olds, and adults. (A) Human data (adapted with permission from page 1523) and (B) REFRESH results.

This dissociation between the age at which good performance on Type I and Type II problems is first displayed can be naturally accounted for by the different component types in REFRESH. Good performance on Type I problems is driven by the singly narrow components, while the Type II advantage is driven by the doubly narrow components. To apply REFRESH to this task, we assumed that adults were using both the singly narrow and doubly narrow covariance components and that *c*_1_ = *c*_2_ = *c*_3_ = 1.5. For these adult parameters, REFRESH showed both fast learning of Type I and a Type II advantage as a result. For all of the groups of children, we assumed the same scale parameters as adults but assumed that only the singly narrow components (i.e., Ψ_*nww*_, Ψ_*wnw*_, and Ψ_*wwn*_) were available. We also assumed, as in the above simulation of dimensional learning, that the variances along the narrow dimension of the singly narrow components were not as narrow as they were for adults. As above, we assumed that it was 0.8 for 3-year olds and 0.5 for 5-year olds. Eight-year olds were not tested above, so here we assumed their narrow standard deviation value was 0.4. The results of this simulation (see Figure 19) match the empirical data in showing that the older children have a marked advantage over the 3-year olds in learning Type I, despite not having much advantage over the 3-year olds in learning the other types. Simulated adults also show the Type II advantage while simulated children do not. However, REFRESH shows an adult advantage for Type IV because performance on this type is tied to performance on the other types through the narrowness in the assumed covariance matrices, while the data do not show an adult advantage on this type. REFRESH also shows an advantage of Type II over Type III, while the adult data, unusually for these kinds of studies, do not. These potential mismatches could be an interesting avenue for future empirical and modeling comparisons.[Fig-anchor fig19]

#### Dimensional Learning

**Figure 20 fig20:**
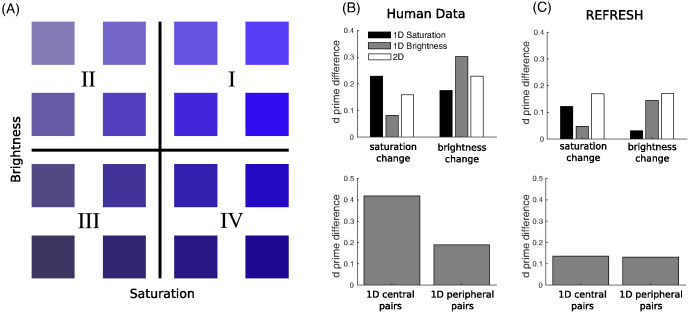
The Effects of Training on Stimulus Discrimination in Experiment 4 of Goldstone (1994) *Note*. (A) Illustration of the type of stimuli used for both categorization training and discrimination. Solid lines are the possible categorization boundaries with the vertical bound used for 1D Saturation training, the horizontal bound used for 1D Brightness training, and both bounds used for 2D Saturation and Brightness training. Quadrants of stimuli are numbered I–IV. (B) Human data from the experiment. The upper plot shows discrimination change measured by difference in *d*′ for each categorization training condition relative to baseline. The lower plot gives the discrimination change for pairs of stimuli that cross the boundary (central) versus pairs that do not (peripheral), averaging across the relevant dimensions of the two 1D training conditions. (C) REFRESH simulations for the same measures as in B. See the online article for the color version of this figure.

As a complement to these developmental results, Goldstone (1994) ran a carefully controlled laboratory demonstration of the effects of categorization training on integral dimensions. In Experiment 4 of that article, participants were given a discrimination task involving 16 stimuli that factorially varied along a pair of integral dimensions: saturation and brightness (see Figure 20A). All participants made same-different judgments between pairs of stimuli that were either both the same or were neighbors. Discrimination ability in this same-different task was measured by the participants’ sensitivity *d*′ to changes on a dimension, and was compared between participants who received categorization training and those who did not. In categorization training, all 16 stimuli were presented 20 times each, and the labels were assigned in three different ways: for 1D Saturation training, all stimuli with below-median saturation (Quadrants II and III) were given one category label and those with above-median saturation were given the other (Quadrants I and IV); for 1D Brightness training, all stimuli with below-median brightness (Quadrants III and IV) were given one category label and those with above-median brightness were given the other (Quadrants I and II); and for 2D Saturation and Brightness training, each quadrant of stimuli was given a different category label.[Fig-anchor fig20]

This experiment tested the extent to which dimensions would compete during learning: Whether participants trained on categories separated along one dimension would improve their discrimination performance along the “relevant” dimension (which was crossed by the category boundary) but reduce it along the “irrelevant” dimension (which was not). This hypothesis follows from the assumption that the Minkowski metric’s dimensional weights sum to one (i.e., ∑_*i*_*w*_*i*_ = 1 in Equation 1). Another prediction of dimensional competition was that 2D Saturation and Brightness training would not improve discrimination performance as much as 1D training along a relevant dimension.

The results of the experiment, shown in Figure 20B, were that training gave a bigger boost to discrimination performance (as measured by *d*′ vs. the baseline of no categorization training) on the relevant dimension compared to the irrelevant dimension for both saturation and brightness. However, there was no competition effect between relevant and irrelevant dimensions: Training also improved performance on the irrelevant dimension. Relatedly, discrimination also improved as a result of 2D Saturation and Brightness training, and there was only weak evidence (statistically significant relative to 1D Brightness but not to 1D Saturation) that training along a single dimension resulted in better discrimination along the relevant dimension than training along both dimensions simultaneously. A final empirical observation was higher discriminability for pairs of stimuli that crossed the boundary than for pairs of stimuli along the relevant dimension that did not cross the boundary, though for both types discrimination was better than it was without training.

We used the untrained version of REFRESH to model these results,^9^ assuming, as for the natural images statistic training, that σ_*r*_^2^ = 1. We drew 1,050 samples from the model via Gibbs sampling and discarded the first 50 samples as burn-in. The remaining samples were then used to determine the probability that each pair of stimuli was the same using the similarity expression in Equation 5, averaging over the two possible assignments of the stimuli to *x* and x*. The *d*′ values were calculated by subtracting the normal inverse of the false alarm rate and from the normal inverse of the hit rate. The simulation was repeated three times, and as each repetition showed the same qualitative results, we averaged over them to produce the model predictions shown in Figure 20C.

REFRESH captures many of the key results, showing increased discriminability for both the relevant and irrelevant dimensions as a result of 1D Saturation and 1D Brightness categorization training. In addition, the discriminability of the relevant dimension increased more than that of the irrelevant dimension. The intuition for the boost to the relevant dimension is that REFRESH learns components that match the trained category structures: they are narrower along the dimension on which the boundary is set. Therefore, stimuli that differ along the relevant dimension, where the expected variance of clusters is smaller, are considered less likely to be part of the same cluster and thus less likely to be judged the same. The intuition for the boost to the irrelevant dimension is that the learned component has a smaller expected variability on the irrelevant dimension than it does prior to training. This explanation also underlies the prediction that 2D Saturation and Brightness training increases discriminability along both dimensions, as was observed empirically. Finally, REFRESH shows a very slightly higher discriminability for pairs of stimuli that crossed the boundary compared to pairs of stimuli that did not cross the boundary.

Not all aspects of the empirical data were reproduced by the model. The overall predicted magnitude of the discriminability increases was smaller than was observed in the data. We could increase the predicted *d*′ values by assuming greater spacing between neighboring stimuli, but for this to be effective a decrease in the dispersion parameter α would likely be needed so that the model does not split each category into many small isotropic clusters. Next, the model’s increased discriminability following 2D Saturation and Brightness training was higher than that for either 1D Saturation or 1D Brightness training, a reversal of the apparent pattern in the data. Finally, there was only a tiny advantage in discriminability for pairs of stimuli that crossed a boundary, which was much smaller than that in the empirical data. This was likely a result of centering the model’s prior in the middle of stimulus space, which is also where each boundary ran. If future empirical work shows that the effect can be found for any boundary, then to match the effect REFRESH would likely have to be augmented to learn of the positions of components in addition to their covariances, which we discuss further in the Limitations and Possible Extensions section.

**Figure 21 fig21:**
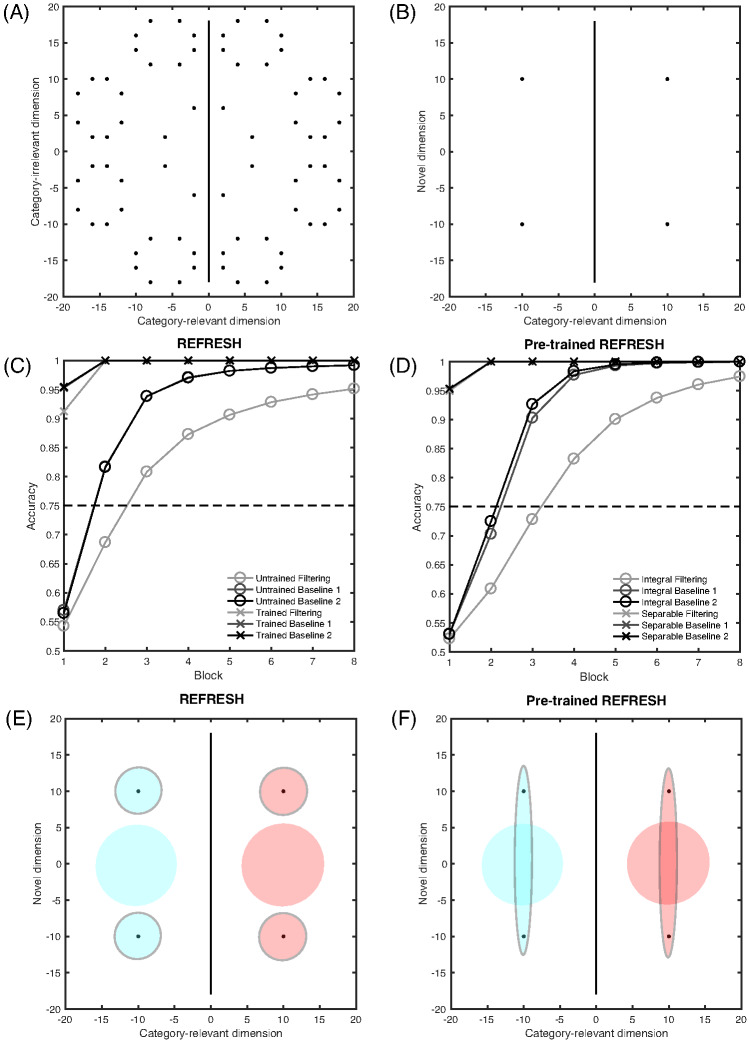
The Effects of Training on Garner Interference in Experiment 1 of Soto and Ashby (2015) *Note*. (A) Illustration of the type of stimuli used for categorization training, with a solid line indicating the categorization boundary. Adapted with permission from page 112. (B) Illustration of the stimuli used in the Garner filtering task. The filtering task asked participants to classify all four stimuli into groups divided by the solid boundary, while the two baseline tasks asked only about either the upper or lower pair of stimuli, so that there was no variation along the novel dimension. Adapted with permission from page 112. (C) REFRESH results for both before and after training for each of the tasks. A performance advantage for the baseline over the filtering task indicates Garner interference. (D) REFRESH results for the pretrained model using either components associated with integral dimensions or those associated with separable dimensions. (E) Cartoon of REFRESH’s clustering and cluster size for the two categories (red and blue) in the filtering task both before (no boundary lines) and after training (gray boundary lines). (F) Cartoon of pretrained REFRESH’s clustering and cluster size for the two categories (red and blue) in the filtering task both for integral (no boundary lines) and separable dimensions (gray boundary lines). See the online article for the color version of this figure.

While the results of Goldstone (1994) are compelling, they do not include the most commonly used test of whether dimensions are separable or integral. This is to use the Garner filtering task (Garner, 1974) to measure how variation along an additional dimension interferes with categorization speed or accuracy, though certainly there are alternative measures that can be deployed (Blunden et al., 2015; Garner, 1974; Little et al., 2013; Soto & Ashby, 2015). In this test, two tasks are compared, using stimuli like those illustrated in Figure 21B. In the filtering task, participants classify stimuli that differ along two dimensions according to where they fall along the target dimension. Performance in the filtering task is subtracted from performance in the baseline task, in which participants classify examples of a pair of stimuli that differ along only the target dimension. A performance advantage in the baseline task is evidence of *Garner interference*—an effect that indicates a pair of dimensions are integral dimensions rather than separable.[Fig-anchor fig21]

Soto and Ashby (2015) investigated whether categorization training reduces Garner interference (Garner, 1974) in their Experiment 1. This task used facial morphs (i.e., pixel-wise averages of photographs of faces) which, unlike saturation and brightness which have in a minority of reports behaved like separable dimensions (Foard & Kemler Nelson, 1984; McKinley & Nosofsky, 1996; Nosofsky, 1987), are novel dimensions for which there is consistent evidence that they are initially integral (Folstein et al., 2012; Goldstone & Steyvers, 2001; Soto & Ashby, 2015). Both the training and test stimuli were defined in separate 2D facial morph spaces, with each dimension defined by a percentage contribution of each of two “anchor” facial photographs. In this study, the dimensions in which the training stimuli and test stimuli were presented changed: The dimension that was relevant for categorization was combined with a novel dimension for test. For the purposes of training and testing REFRESH, we trained and tested in a 3D space that included the variation of both the training and test stimuli, assuming that when a dimension was not in use that all values along that dimension were zero, reflecting zero contribution of that dimension’s anchor faces to the resulting morph. This is of course a rough approximation to the psychological representation of faces. An illustration of the parameter values of the training stimuli is shown in Figure 21A.

Soto and Ashby (2015) found that categorization training increased overall performance and reduced Garner interference. The reduced interference held for both response times and accuracy as well: The average accuracy interference was 3.3% for participants without training and 0.2% for participants given categorization training. As REFRESH does not provide an account of response times, we only attempted to reproduce the accuracy differences. As in Soto and Ashby (2015), we trained the untrained REFRESH on nine blocks, each consisting of all of the stimuli in Figure 21A in the categorization condition, using the same parameters and simulation details as for the Goldstone (1994) experiment. We selected 10 samples (choosing every 10th sample after discarding the first 50 of 150 total samples) of the component parameter posterior distributions and for each sample calculated the modal covariance matrices, then used these in the particle filter approximation to determine classification performance. The block-by-block accuracy results are shown in Figure 21C, demonstrating that before training there was Garner interference and that training increased performance and greatly reduced interference. Soto and Ashby (2015) summarized interference by averaging the classification error rate for eight blocks of the filtering task and four blocks each of the two possible baseline tasks, but only for blocks in which the proportion of correct responses exceeded 75%. Calculating interference in the same way, we find the same qualitative pattern but with smaller magnitudes compared to the empirical data, and the results shown in Figure 21C are clearly robust to other ways of summarizing performance.

The intuition for REFRESH’s reduction of Garner interference is that categorization training shrunk the expected “size” of clusters. In the untrained condition, there was a separate cluster for each stimulus in the baseline task, but in the filtering task at times both stimuli with the same label were assigned to the same cluster, which reduced classification accuracy. After training, the reduced cluster size meant that in the filtering condition each stimulus was more likely to be assigned to its own cluster which resulted in near-perfect classification accuracy, as also found in the baseline task (see Figure 21E). This ceiling performance was a consequence of needing to choose a spacing between neighboring stimuli (doubled from that in Figure 21B) that allowed performance in the untrained conditions to eventually exceed the 75% threshold. We also performed a secondary analysis that reduced the distance between stimuli to 5% of what it was originally to bring the trained condition off of the ceiling, which resulted in chance-level performance in the before training condition. In this secondary analysis, there was still very little Garner interference in the trained condition.

This, however, is not the only way in which REFRESH could produce the usual empirical patterns in Garner interference. We also investigated the performance of the trained model on the exact same experimental design. For this simulation, we assumed that there were two components for separable dimensions, Ψ_*nww*_ and Ψ_*wwn*_, which meant that for the two dimensions along which there was variability for the testing stimuli, the available components reduced to Ψ_*nw*_ and Ψ_*wn*_. For integral dimensions, only the isotropic component, Ψ_*www*_, was available. For all dimensions *c*_*d*_ = 0.3 in this simulation. The results of this simulation are shown in Figure 21D, which look very similar to those in Figure 21C. Again, this plot shows ceiling performance for the separable dimensions, and so in a secondary analysis we set all *c*_*d*_ = 0.03 to bring separable dimension performance off of the ceiling, which necessitated bringing integral dimension performance to the floor. This secondary analysis showed very little Garner interference for separable dimensions.

However, despite the similarity in performance, the reduction in Garner interference with training for separable dimensions occurs for a different reason: The shape of the expected covariance matrix is elongated in our separable dimensions model. As a result, stimuli with the same label are put into the same cluster in the filtering task (which is the cause of interference in the modeling above), but the narrowness of the distribution greatly reduced interference (see Figure 21F). Interestingly, this second way in which REFRESH can produce the empirical pattern of Garner interference does not correspond functionally with a better ability to ignore an irrelevant separable dimension than an irrelevant integral dimension because the variances along those dimensions are the same. Instead, it corresponds to a greater ability to stretch the relevant separable dimension, which is an alternative hypothesis that would be interesting to explore in future training studies.

## The Origin of Dimensional Biases

Like the exemplar and prototype models, REFRESH uses as its basis the concept of a continuous psychological space. This concept has been very useful in many theories of similarity and generalization, because it provides a natural way to generalize from observed to novel objects by utilizing their proximity in psychological space. This continuous space can be seen as reflecting the continuous parameterizations of aspects of objects such as position and motion, color, and representations of object kind in the outside world (Shepard, 1994).

There have been a number of hypotheses about how separable and integral dimensions are formed in a psychological space. As discussed above, Shepard’s influential account of similarity and generalization within a psychological space holds that they are the result of inferring how likely two stimuli are of the same natural kind, meaning that they belong to the same consequential region of the psychological space. Formally, the similarity between two stimuli is thought to be the probability that the relevant consequential region that contains one stimulus also contains the second stimulus. However, the size and shape of the consequential region are unknown, so generalization requires integrating over all of the possible consequential regions, weighting each by its prior probability (Tenenbaum & Griffiths, 2001a). This scheme naturally produces the kinds of exponential and Gaussian generalization functions seen empirically in the psychological space (Ennis & Shepard, 1988; Shepard, 1987) and can be adapted to fit human categorization data quite well (Shanks & Gluck, 1994). To explain the origin of dimensional biases, Shepard (1991) argued that integral dimensions were the result of a positive correlation in stimulus variability along these dimensions, while separable dimensions were the result of zero correlation in stimulus variability along these dimensions.

A developmental theory of how separable dimensions are formed in a psychological space was proposed by Smith and Kemler (1978). Initially for children, dimensions that are separable for adults are *nonprimary axes*, meaning that the stimuli are not perceived as showing differences along any particular dimension, and any rotation of the axes would provide an equally good description of the stimuli. As children become older, these adult-separable dimensions become *primary axes*, which are accessible to older children with effort, but they are not immediately available. Finally, adult-separable dimensions are *obligatory axes* in that they are available immediately and without effort and cannot be ignored. Smith and Kemler (1978) hypothesized that all pairs of dimensions follow this developmental trajectory, though at different rates and with some pairs progressing further than others.

REFRESH can be viewed as a formal extension of both of these ideas. REFRESH uses graded consequential regions instead of the all-or-none regions introduced by Shepard, but like this theory it involves uncertainty about the parameters of the region that are integrated over in order to produce the generalization gradients. Moreover, Shepard (1987, 1991) hypothesized that separable and integral dimensions were driven by the same correlations in the variance of categories along pairs of dimensions that we empirically observed in natural image statistics (see Figure 9). These statistics were then used as inspiration for our choice of priors in the trained model.

When REFRESH is trained on these natural image statistics, it goes through a similar progression to that hypothesized by Smith and Kemler (1978). Its initial prior treats the space as having nonprimary axes in which the similarity gradient can be rotated without any effect on performance (see Figure 11 with zero clusters), and with a large amount of training this initial prior will be overwhelmed by the learned components which will behave like obligatory axes. The intermediate stage of nonprimary axes may possibly map onto REFRESH with a moderate amount of training: while there is still an overall isotropic prior over components, several weakly axis-oriented components have been learned, and there is uncertainty about which will better describe the observed stimuli. A difference is that REFRESH does not have a mechanism for cognitive effort to act to make primary axes separable as it is a computational-level model, but we revisit this in the Rational Process Models section below.

What both of these proposals were lacking however was a means by which to explain which dimensions, out of an infinite set of possible rotations of the dimensions of the space, would become separable dimensions. The advantage of using REFRESH’s rich hierarchical prior is that it allows us to avoid explicitly learning the preferred axes of the psychological space: the separable dimensions that are associated with dimensional biases. The infinite number of possible orthogonal preferred axes presents a substantial learning challenge. Compounding this problem is evidence that representations are not necessarily orthogonal, as has been found in the perception of rectangles (Krantz & Tversky, 1975; Macmillan & Ornstein, 1998). Our approach allows for nonorthogonal dimensional biases, and removes the need for an additional complex learning mechanism for determining the preferred axes.

A final empirical hurdle that has previously made it difficult to design a mechanism for learning the preferred axes is that these axes depend not only on the distribution of the stimuli, but also on the structure of categories (Austerweil & Griffiths, 2013; Austerweil et al., 2019; Schyns et al., 1998). This means that unsupervised learning based on image statistics, such as Principal Components Analysis (PCA) which finds the set of orthogonal dimensions that capture the ways in which the stimuli vary the most, will not by itself produce a representation that matches human categorization.

Two existing approaches already address, to some extent, dimensional learning using category information. The first, known as category packing, assumes that people attempt to create categories that both maximize classification accuracy while at the same time minimizing overlap with other categories. Because different kinds of stimuli, such as solid and nonsolid objects, are organized into categories along different dimensions, new categories that attempt to minimize overlap with previous categories will also take on the same kind of local organization (Hidaka & Smith, 2011; see also Conway & Austerweil, 2017). This model is in some ways similar to REFRESH, and has the added advantage of predicting shape and material biases, but it has two major limitations. The first limitation of this approach is that it has no mechanism for predicting trial-by-trial category judgments, like those required in the condensation versus filtration or the SHJ Type II advantage experiments, which is why we did not review it above. The second limitation is that, like the hierarchical Bayesian model of categorization proposed by Kemp et al. (2007), it effectively only learns to generalize along a single separable dimension for each stimulus rather than learning a set of separable dimensions. For example, if this model learns that shape is an important dimension for determining the similarity to a stimulus and that color is unimportant, it cannot also learn that sometimes it is important to consider color and disregard shape for that same stimulus. As a result, even with a mechanism for predicting trial-by-trial category judgments, it could not fully produce the classic dimensional biases, as it is necessary for individual stimuli to display dimensional biases for multiple dimensions, not just a single dimension.

A second approach, presented in Colunga and Smith (2005), is an associative learning model based on a Hopfield network that can learn from experience with past categories how to generalize to new stimuli differently. Similar to the category packing approach, it can learn the correlation between category structure and solid and nonsolid objects, which REFRESH cannot. This model included a mechanism for translating the similarities of the internal representation into a judgment of which item of a pair belonged with a standard, which is insufficient for predicting trial-by-trial category judgments when the categories consist of more than one item. Because this model’s representation is opaque, it is unclear whether it would be able to learn a *set* of separable dimensions that can take different weights within the stimulus space, as REFRESH can. However, it seems less important to directly test this associative learning model than addressing the more general claim made by Colunga and Smith (2005; see also Goldstone, 2003; Spratling, 2006; Spratling & Johnson, 2006): that a connectionist model can learn regularities across categories. Given the success that more complex deep networks have had in learning regularities across categories (Vinyals et al., 2016), we believe that there is almost certainly some kind of connectionist network that can learn from the statistics of natural categories as REFRESH can. While we could have presented a connectionist model in this work, we chose to develop a Bayesian model because its predictions derive more clearly from the structure of the environment, rather than the algorithmic implementation of the model.

Of course, even if dimensional biases are learnable from the structure of the environment, as we have argued, these biases may still be innate rather than learned, and come to the fore over development (e.g., Smith, 1989). This is a certainly a possibility for some dimensions that we have investigated such as size and color, as they were likely to have been useful dimensions throughout human evolution. And it may even explain the dimensional biases of some artificial stimuli, like “Shepard circles,” which are not generally present in the natural environment but can appear separable in the responses produced by Gabor filters (Tijsseling & Gluck, 2002). But it is unlikely to explain how dimensions can be trained to be separable using arbitrary dimensions in facial morph spaces (Soto & Ashby, 2015). Considering all these types of separable dimensions, it is plausible that at least some separable dimensions are learned through an individual’s lifetime of experience, rather than being latently available, and an explanation of how this could work is REFRESH’s main contribution.

## Limitations and Possible Extensions

It is unlikely that any single model can fully explain the complexity and variety of human categorization, which needs to capture abstract categories like space, number, responsibility, the good, and so on, and the variety of influences on such categorization, which in principle are shaped by world-knowledge of just about any kind. Above, we took a particular approach to categorization, that of a pure family resemblance model that learns across categories and asked what aspects of categorization it can capture. To do so, we used a set of parameters in the trained model that was inspired by natural image statistics but not derived directly from them. Further investigating environmental statistics and forging a tighter link to model parameters is one important avenue for future work. Below, we look at a broader set of desiderata for a categorization model and discuss the ways in which REFRESH could be extended or modified to account for them.

### Shape and Material Biases

In our initial discussion of the role of learning in dimensional biases above, we briefly discussed children’s bias to extend words to objects of the same shape for solid objects, and to extend words to objects of the same material for nonsolid objects. We also discussed how these biases also seem to reflect the structure of categories in the world.

Training studies with children provide the best evidence that the stimulus-dependence of dimensional biases is learned. Children trained on named categories organized by shape were able to generalize this regularity to other categories. This training had an effect on later word learning: Trained children learned nouns faster outside of the laboratory than children not given this training (Smith et al., 2002). Training on categories organized by shape only helped with categories organized by shape; children trained in this way also overgeneralized the shape bias to nonsolid objects, and children who were trained with categories constant in material showed less overgeneralization (Samuelson, 2002). Additionally, children can be trained to generalize differently for animate and inanimate categories: generalizing along shape alone for inanimate objects and generalizing along shape and material for animate objects (Jones & Smith, 2002).

REFRESH, as it is formalized above, cannot produce biases of this kind, because this dimensional bias does not just depend on the dimensions chosen or the spacing between stimuli, but also on the actual values of the stimuli along the chosen dimensions. In studies of the shape bias, participants are given an object and asked to choose which of two other objects shares the same label: An object with the same shape but different material from the target object, or an object with a different shape but same material. Adults are biased toward choosing the object with the same shape if the objects share a complex shape, but are much less likely to do so if the objects are blobs of material (Soja et al., 1991). However, it is straightforward to extend REFRESH to explain these effects by allowing components to have different means (which are learned) and by assuming the kinds of category structures found empirically by Samuelson and Smith (1999): that categories of solid stimuli tend to be consistent in shape, while categories of nonsolid stimuli tend to be consistent in material. This kind of extension could potentially also explain the greater sensitivity to stimuli that cross a learned decision boundary observed in the experiments of Goldstone (1994), as the components that allow for greater sensitivity will be more influential closer to where they were trained.

### Feature Learning

REFRESH views categories as a composition of clusters of objects, and could potentially learn that different components describe clusters in the same category. How does this compare to models of feature learning? One very relevant model, presented in Austerweil and Griffiths (2011), could learn the features of an object using a nonparametric prior described in terms of pixel values. One key difference between this model and REFRESH is in their assumptions regarding the latent structure an object can have: either clusters or features. An object in REFRESH is assigned to exactly one cluster, whereas an object in their model is assigned zero or more features. This results in different expectations about likely novel objects. For example, REFRESH does not expect a novel object in which half of the object’s dimensions come from one cluster and the other half of its dimensions come from another cluster, while their model would expect such an object.

Some of the empirical results presented by Austerweil and Griffiths (2011), as support for their model could plausibly be captured by REFRESH. For example, they effectively distinguished their model from exemplar and prototype approaches in Experiment 2, which presented participants with a set of objects that shared a novel property. Participants were then asked to generalize the novel property to new examples. The exemplar and prototype models generalized from the previous examples but were misled by the noise added to the images. REFRESH could potentially avoid this trap because it could distinguish between the pixels that are constant and those that are variable (i.e., the noisy pixels) and would not rely on the noisy pixels when generalizing.

Later work by the same authors formulated two schemes for incorporating category information into their approach (Austerweil & Griffiths, 2013). One, like the RMC, treats category labels as equivalent to pixel values and learns a set of features that can encode both pixel values and category labels. In this scheme, if certain sensory inputs are correlated enough with a category label, a single feature will encode them together. Their second scheme for incorporating category information treats the category of an object differently from the object’s sensory data. In the second scheme, there is one central repository of features, and the features for each category are separately sampled from that repository. In both schemes, objects are treated differently than in REFRESH because each object is encoded by zero or more features, and potentially could have features from multiple categories, whereas REFRESH encodes each object with a single cluster and so assumes it is generated from a single category.

Rather than viewing REFRESH and feature learning models as rival models of categorization, we believe that they describe different assumptions about how features or clusters are used to represent the set of possible objects in a category. The models could potentially be dissociated by forming object sets that adhere to either the assumptions of REFRESH or the feature learning model. Both can be sensible assumptions in certain environments, and we suspect we would find that each model captures human performance when the objects given to people adhere to the assumptions of that model. However, we do not believe this is the best way forward. Instead, it seems most promising to combine the two approaches, so the features estimated by the feature learning model serve as inputs to REFRESH. Combining the two approaches would result in a model able to make predictions about how previous categorization experience biases the features that are learned, potentially providing a route to explaining interactions between categorical and perceptual constraints in feature extraction (Schyns & Murphy, 1994).

### Rational Process Models

We presented REFRESH as a computational-level model, as it describes the statistical problem to be solved (Marr, 1982), though we also noted that algorithmic-level constraints might be necessary to justify some of our parameter settings. However, it is reasonable to expect that the brain, with its limited capacity, would be unable to implement the statistical model exactly. REFRESH requires the representation of an enormous number of hypotheses: The representation of all possible partitions of objects we have experienced into clusters, crossed with all possible partitions of clusters into components. Even representing the possible partitions of a lifetime of experienced objects into clusters is an impossible task, as just 100 experienced objects would require 4.7 × 10^115^ possible partitions to be represented—a number far greater than the number of atoms in the observable universe. As a consequence, the model must be approximated for all but the smallest collections of objects.

For our simulations, we used a set of approximations developed in computer science and statistics (see Appendix), and we can explore whether these same approximations might also be used by the brain. This is the approach of creating *rational process models*: combine a rational model with an approximation algorithm that makes sense for the particular task, fits the behavioral data, and ideally satisfies known psychological and neural constraints (Griffiths et al., 2012).

Because real-world categories are learned through experience slowly over time, and people need to make category judgments as they are learning, it is sensible to focus on algorithms that work well for data that arrive sequentially. A commonly used set of algorithms for sequential data are particle filters, which are a family of algorithms that modify a set of samples from a prior distribution so that they reflect the probabilities of a posterior distribution. Particle filters have been used successfully in rational process models of online tasks such as change detection (Brown & Steyvers, 2009), problem-solving (Yi et al., 2009), sentence parsing (Levy et al., 2009), and learning (Abbott & Griffiths, 2011; Daw & Courville, 2008), as well as in categorization tasks (Austerweil & Griffiths, 2013; Sanborn et al., 2010). Neurally plausible versions of particle filters have been developed, providing a potential link to neural data (Huang & Rao, 2014; Lee & Mumford, 2003; Legenstein & Maass, 2014).

In categorization tasks, particle filters have been used to explain how the learned category representation depends on the order in which the stimuli are presented. Order effects are not predicted by most Bayesian models of categorization, including REFRESH, because these models are stationary: they assume categories do not change over time. However, particle filters can produce order effects even with stationary models because of the way they update the prior samples to reflect the posterior. Essentially, when the posterior is very different from the prior, it is difficult to modify the prior to match the posterior. The order effects that occur are then those that result from the algorithm going down a “garden path”: some hypotheses may seem very plausible based on the initial data, but when later data greatly change what are the best hypotheses, the particle filter may not be able to overcome its initial bias in what it represents. Order effects that were empirically observed in free categorization (Anderson, 1990) and feature learning (Schyns & Rodet, 1997) can both be explained in this way (Austerweil & Griffiths, 2013; Sanborn et al., 2010).

Recent work has used the number of particles to explain the link between working memory capacity (WMC) and categorization performance. More particles result in faster learning because the hypothesis that best explains new data is more likely to already be represented in the prior, so it will be easier for the particle filter to “discover” this hypothesis. In experimental studies, WMC has been linked both to better overall categorization performance (Lewandowsky, 2011) and to a greater ability to restructure categorization knowledge when participants are asked to focus on different cues (Sewell & Lewandowsky, 2012). A particle filter implementation of a different Bayesian model of categorization can explain both of these effects with a single mechanism: assuming that WMC is related to the number of particles that an individual has available to represent the posterior distribution (Lloyd et al., 2019).

**Figure 22 fig22:**
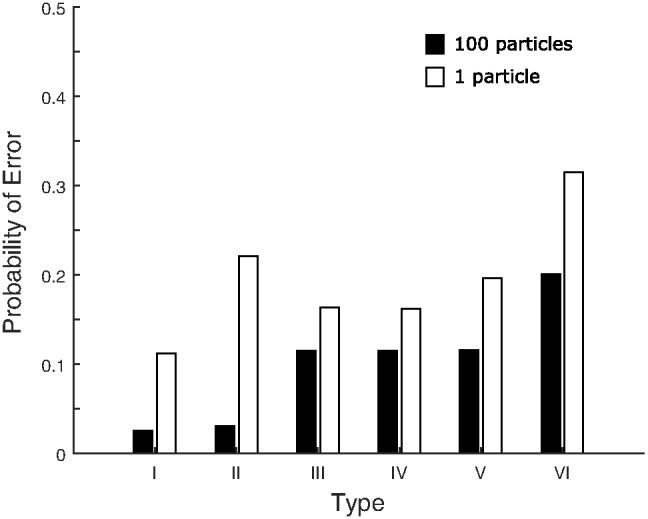
Rational Exclusively Family RESemblance Hierarchy Predictions for how Errors for the Six Shepard et al. (1961) Problem Types Depend on Inferential Resources: The Number of Particles Used in a Particle Filter

Using a particle filter to perform inference in our model could explain how WMC impacts learning in the SHJ problems. A reanalysis of the data of Lewandowsky (2011) by Lloyd et al. (2019) showed that higher WMC results in a larger advantage of Type II over Type IV. There is some evidence that the ordering can even reverse: comparisons of older and younger adults show that older adults, who have worse working memory generally, perform better on Type IV than Type II problems (Badham et al., 2017; Rabi & Minda, 2016). We can match this result by decreasing the number of particles in REFRESH (see Figure 22): For one hundred particles Type II is easier than Type IV, but for one particle the ordering reverses. This provides another interesting link between WMC and this inferential resource.[Fig-anchor fig22]

Using a particle filter for inference in REFRESH could also potentially explain empirical effects that seemingly argue against a family resemblance representation. Across a variety of studies, participants seem to use a single dimension at a time to make their categorization judgments, at least early in learning (e.g., Nosofsky, Palmeri, et al., 1994; Smith et al., 2014), and the transitions between using different single dimensions appear to be sudden (Smith & Ell, 2015). This intuitively looks more like a search for a correct rule than the operation of a family resemblance model, but REFRESH may be able to produce this effect as a result of using approximate inference. Using only a small number of samples to represent hypotheses about which prior component applies to each cluster could lead to only a single component being represented at times. It could also lead to sudden transitions as the small number of particles switch the assignment of a cluster to a different component. Cognitive effort could be modeled as the amount of additional sampling needed to find hypotheses that are a priori unlikely yet very useful in a particular problem, potentially explaining the effort needed to make primary axes separable.

While using a particle filter for inference in REFRESH could both make the model more tractable as well as better match human data, additional constraints may need to be incorporated. For example, using a particle filter would not impose any kind of capacity limit on the covariances used in the trained version of REFRESH, which as discussed above seems to be necessary to fit a range of human data. It may be that there are additional implementation constraints and capacities, such as trade-offs in attention between dimensions, that need to be taken into account in a rational process version of REFRESH (e.g., Lieder & Griffiths, 2020).

### Explaining Evidence for Multiple Systems With a Single System

An active debate within the field of categorization is whether category learning is performed by a single system or multiple systems. In particular, proponents of the dual-system model COVIS point to 20 years’ worth of empirical results that dissociate the two systems in this model: an explicit rule-learning system, and an implicit procedural system (see Ashby & Valentin, 2017, for a review). REFRESH is a single system model, and here we discuss how well it might account for these dissociations, though as we stated at the beginning of the section, we do not expect that REFRESH will be able to account for every effect in the field.

To dissociate the two systems in COVIS, two types of categorization tasks are used: a rule-based (RB) task and an information-integration (II) task. These tasks are very similar to the condensation and filtration tasks (see Figure 2) we modeled above. In RB tasks, participants learn to classify stimuli with two continuous parameters, such that the classification can be made by looking at only one of the parameters, like in the filtration task. This task can be performed perfectly using COVIS’ explicit rule-learning system. In contrast, II tasks require participants to classify stimuli using both perceptual dimensions at one time, like in the condensation task. II tasks cannot be performed perfectly by the explicit rule-learning system and instead the implicit procedural system is required to do so.

Applying the same manipulation to RB and II tasks often results in dissociations in performance. For example, when feedback is removed, II performance suffers a decrement, but RB performance is unchanged (Ashby et al., 1999). Further work has manipulated both the proportion of II trials that received feedback and also the total number of II training trials that participants were given, and successful learning of the II task was found to depend on the number of feedback trials that participants received, rather than on the number of no-feedback trials (Vandist et al., 2009; cf., Ashby & O’Brien, 2007).

This dissociation could potentially be explained by a computational-level model such as REFRESH because these experimental manipulations impact the information available to participants. As the amount of information available via feedback is reduced, REFRESH will rely more heavily on its priors learned through experience with other categories. These prior components are aligned with the separable dimensions used in these studies, matching the RB task better than the II task. Simply because the II task is less like the model’s prior, reducing feedback will reduce performance on this task more. This route could also be used to explain the same dissociation with a related manipulation: When feedback is not given on a trial-by-trial basis, but instead summarized at the end of a series of trials so that participants are only aware of their general performance on a block (Smith et al., 2014). Summarized feedback does not allow participants to know which trials were incorrect and so the loss of information would likely impact the II task more than the RB task in our model, as was found empirically.

There are, of course, a variety of dissociations found using RB and II tasks that work in the opposite direction: Learning on the RB task is affected but learning on the II task is not. Many of these manipulations are aimed to occupy or deplete cognitive resources in one way or another, such as giving a concurrent task that engages working memory (e.g., Zeithamova & Maddox, 2006). Above we discussed how WMC can be modeled as an inferential resource such as the number of particles in a particle filter. While we do not attempt to discuss in detail the results here, relevant to the question of whether REFRESH could fit these data are analyses of whether concurrent working memory load reveals a single process or multiple processes at work. There is no consensus at present (Ashby, 2014, 2019; Newell et al., 2010; Stephens et al., 2020), but we note that it has also been shown that an individual’s WMC helps determine performance on both RB and II tasks (Lewandowsky et al., 2012).

A strength of COVIS is that it is also able to explain a variety of other dissociations that occur in specific patient populations that are beyond the scope of REFRESH (reviewed in Roeder et al., 2017). These dissociations have been predicted by COVIS because of the mapping of its two systems onto different areas of the brain. Our model would likewise need a neural implementation in order to test whether it can explain these dissociations, which we do not attempt to specify here. This future work could look to the successful theories of how COVIS is implemented for inspiration, perhaps by separately mapping different parts of our single process model to different regions of the brain.

## Conclusions

In almost all accounts of human categorization, separable dimensions are the primitives generated by the perceptual system that the categorization system can then use in various ways, perhaps using them to construct rules or perhaps using them to modify distances in a psychological space. The model we present here, REFRESH, takes a very different view of separable dimensions. While the dimensions continue to exist in the psychological space, they no longer have any special status and are not used as primitives. Taking this novel view of separable dimensions allowed REFRESH to not only explain the classic dimensional biases, but also their stimulus-dependence and how they are learned. While our account argues for the diminished importance of separable dimensions, it gives them greater purpose: Dimensional biases are the result of the categorization system adapting itself to the environment.

## Figures and Tables

**Table 1 tbl1:** Summary of Dimensional Biases in Perceptual Similarity and Categorization

Type	Effect name	Effect description	Example citation(s)
The classic dimensional biases	Violations of the triangle inequality	Similarities for some dimensions violate the requirements of the metric space used for family resemblance	Tversky and Gati (1982)
	Condensation vs. filtration	Dimension-aligned categories are easier to learn than misaligned categories	Garner (1974) and Kruschke (1993)
	SHJ Type II advantage	Exclusive-or categories are easier to learn than family resemblance predicts	Shepard et al. (1961)
Stimulus dependence of dimensional biases	Separable vs. integral dimensions	Pairwise nature of separable or integral dimensions; filtration advantage and SHJ Type II advantage disappear with integral dimensions; biconditional discrimination is easier for integral dimensions	Gottwald and Garner (1975), Nosofsky and Palmeri (1996), and Soto et al. (2015)
	SHJ Type II advantage dependencies	SHJ Type II advantage only for some separable dimensions	Love and Markman (2003) and Kurtz et al. (2012)
Learning dimensional biases and their stimulus dependence	Dimensional development	Dimensional generalization develops; young children can learn simple categories like adults but only older children show an adult-like advantage for exclusive-or categories	Smith (1989) and Minda et al. (2008)
	Dimensional learning	Categorization training increases perceptual discrimination; integral dimensions can be trained to be separable dimensions	Goldstone (1994) and Soto and Ashby (2015)

**Table 2 tbl2:** Comparing Categorization Models Ability to Produce the Dimensional Biases in Table 1

Effect type	Effect name	Rules (RULEX, rational rules)	Exemplar (GCM, ALCOVE)	Hybrid (ATRIUM, COVIS)	Rational model of categorization	Hierarchical rational models	REFRESH
The classic dimensional biases	Violations of the triangle inequality	✓	✓	✓		✓	✓
	Condensation vs. filtration	✓	✓	✓	✓	✓	✓
	SHJ Type II advantage	✓	✓	✓	✓	✓	✓
Stimulus dependence of dimensional biases	Separable vs. integral dimensions		✓	✓		✓	✓
	SHJ Type II advantage dependencies	✓	✓	✓		✓	✓
Learning dimensional biases and their stimulus dependence	Dimensional development	✓	✓	✓		✓	✓
	Dimensional learning						✓
*Note*. REFRESH = Rational Exclusively Family RESemblance Hierarchy, RULEX (Nosofsky, Palmeri, et al., 1994), GCM (Nosofsky, 1986), ALCOVE (Kruschke, 1992), ATRIUM (Erickson & Kruschke, 1998), and COVIS (Ashby et al., 1998).							

**Table A1 tbl3:** Covariance Matrices Used in the Trained REFRESH Approximation

Dimensions	Type	Covariance matrices
Two	Isotropic	$\Psi_{ww}=(\begin{array}{cc} {\left(\frac{1}{c_{1}}\right)}^{2} & 0\\ 0 & {\left(\frac{1}{c_{2}}\right)}^{2} \end{array})$
	Singly narrow	$\Psi_{nw}=(\begin{array}{cc} {\left(\frac{0.1}{c_{1}}\right)}^{2} & 0\\ 0 & {\left(\frac{1}{c_{2}}\right)}^{2} \end{array}),\;\Psi_{wn}=(\begin{array}{cc} {\left(\frac{1}{c_{1}}\right)}^{2} & 0\\ 0 & {\left(\frac{0.1}{c_{2}}\right)}^{2} \end{array})$
Three	Isotropic	$\Psi_{\mathrm{www}}=(\begin{array}{ccc} {\left(\frac{1}{c_{1}}\right)}^{2} & 0 & 0\\ 0 & {\left(\frac{1}{c_{2}}\right)}^{2} & 0\\ 0 & 0 & {\left(\frac{1}{c_{3}}\right)}^{2} \end{array})$
	Singly narrow	$\Psi_{\mathrm{nww}}=(\begin{array}{ccc} {\left(\frac{0.1}{c_{1}}\right)}^{2} & 0 & 0\\ 0 & {\left(\frac{1}{c_{2}}\right)}^{2} & 0\\ 0 & 0 & {\left(\frac{1}{c_{3}}\right)}^{2} \end{array}),\;\Psi_{\mathrm{wnw}}=(\begin{array}{ccc} {\left(\frac{1}{c_{1}}\right)}^{2} & 0 & 0\\ 0 & {\left(\frac{0.1}{c_{2}}\right)}^{2} & 0\\ 0 & 0 & {\left(\frac{1}{c_{3}}\right)}^{2} \end{array}),\;\Psi_{\mathrm{wwn}}=(\begin{array}{ccc} {\left(\frac{1}{c_{1}}\right)}^{2} & 0 & 0\\ 0 & {\left(\frac{1}{c_{2}}\right)}^{2} & 0\\ 0 & 0 & {\left(\frac{0.1}{c_{3}}\right)}^{2} \end{array})$
	Doubly narrow	$\Psi_{\mathrm{wnn}}=(\begin{array}{ccc} {\left(\frac{1}{c_{1}}\right)}^{2} & 0 & 0\\ 0 & {\left(\frac{0.3}{c_{2}}\right)}^{2} & 0\\ 0 & 0 & {\left(\frac{0.3}{c_{3}}\right)}^{2} \end{array}),\;\Psi_{\mathrm{nwn}}=(\begin{array}{ccc} {\left(\frac{0.3}{c_{1}}\right)}^{2} & 0 & 0\\ 0 & {\left(\frac{1}{c_{2}}\right)}^{2} & 0\\ 0 & 0 & {\left(\frac{0.3}{c_{3}}\right)}^{2} \end{array}),\;\Psi_{\mathrm{nnw}}=(\begin{array}{ccc} {\left(\frac{0.3}{c_{1}}\right)}^{2} & 0 & 0\\ 0 & {\left(\frac{0.3}{c_{2}}\right)}^{2} & 0\\ 0 & 0 & {\left(\frac{1}{c_{3}}\right)}^{2} \end{array})$
*Note*. REFRESH = Rational Exclusively Family RESemblance Hierarchy.		

**Table A2 tbl4:** Summary of REFRESH Parameter Values

Effect produced	Figure ref.	α	α_*c*_	α_*g*_	*v*_*t*_	σ_*r*_^2^	Approximation	Covariances	*c*_*d*_
Learning from image statistics	Figure 10	10	0.001	1	30	1	Gibbs	N/A	N/A
Triangle inequality violations	Figure 11	10	0.001	1	30	1	Gibbs	N/A	N/A
Condensation vs. filtration	Figure 12C	10	0.001	0	N/A	0.1	Particle filter	Ψ_*nw*_, Ψ_*wn*_	*c*_1_ = *c*_2_ = 0.5
Condensation vs. filtration	Figure 12D	10	0.001	0	N/A	0.1	Particle filter	Ψ_*nw*_, Ψ_*wn*_	*c*_1_ = 0.5, *c*_2_ = 1
Condensation vs. filtration	Figure 12E	10	0.001	0	N/A	0.1	Particle filter	Ψ_*ww*_	*c*_1_ = *c*_2_ = 2
SHJ Type II advantage	Figure 13D	10	0.001	0	N/A	0.1	Particle filter	Ψ_*nww*_ – Ψ_*nnw*_	*c*_1_ = *c*_2_ = *c*_3_ = 2.1
SHJ Type II advantage	Figure 13E	10	0.001	0	N/A	0.1	Particle filter	Ψ_*www*_	*c*_1_ = *c*_2_ = *c*_3_ = 2.5
SHJ Type II advantage	Figure 13F	10	0.001	0	N/A	0.1	Particle filter	Ψ_*nww*_ – Ψ_*wwn*_	*c*_1_ = *c*_2_ = *c*_3_ = 2.5
Biconditional discrimination	Figure 16C	10	0.001	0	N/A	0.1	Particle filter	Ψ_*wwn*_, Ψ_*nnw*_	*c*_1_ = *c*_2_ = *c*_3_ = 1.3
SHJ Type II dependencies	Figure 17C	10	0.001	0	N/A	0.1	Particle filter	Ψ_*nww*_ – Ψ_*nnw*_	*c*_1_ = 0.7, *c*_2_ = *c*_3_ = 2.3
SHJ Type II dependencies	Figure 17D	10	0.001	0	N/A	0.1	Particle filter	Ψ_*nww*_ – Ψ_*nnw*_	*c*_2_ = 0.7, *c*_1_ = *c*_3_ = 2.3
Dimensional development	Figure 18B	1	0.001	0	N/A	100	Exact partition	Ψ_*nw*_^*a*^, Ψ_*wn*_^*a*^	*c*_1_ = *c*_2_ = 2.3
SHJ development (adults)	Figure 19B	10	0.001	0	N/A	0.1	Particle filter	Ψ_*nww*_ – Ψ_*nnw*_	*c*_1_ = *c*_2_ = *c*_3_ = 1.5
SHJ development (children)	Figure 19B	10	0.001	0	N/A	0.1	Particle filter	Ψ_*nww*_^*a*^ − Ψ_*wwn*_^*a*^	*c*_1_ = *c*_2_ = *c*_3_ = 1.5
Training and discrimination	Figure 20C	10	0.001	1	30	1	Gibbs	N/A	N/A
Training and Garner interference	Figure 21C	10	0.001	1/0^b^	30^c^	1	Gibbs then particle filter	N/A	N/A
Training and Garner (integral)	Figure 21D	10	0.001	0	N/A	0.1	Particle filter	Ψ_*www*_	*c*_1_ = *c*_2_ = *c*_3_ = 0.3
Training and Garner (separable)	Figure 21D	10	0.001	0	N/A	0.1	Particle filter	Ψ_*nww*_, Ψ_*wwn*_	*c*_1_ = *c*_2_ = *c*_3_ = 0.3
*Note*. REFRESH = Rational Exclusively Family RESemblance Hierarchy; N/A = not applicable. Ψ_*nww*_ – Ψ_*nnw*_ refers to the collection Ψ_*nww*_, Ψ_*wnw*_, Ψ_*wwn*_, Ψ_*nnw*_, Ψ_*nwn*_ and Ψ_*wnn*_, while Ψ_*nww*_ – Ψ_*wwn*_ refers to the collection Ψ_*nww*_, Ψ_*wnw*_, and Ψ_*wwn*_.									
^a^ The standard deviations equal to 0.1 in these covariance matrices used for adults were replaced with 0.4 for 8-year olds, 0.5 for 5-year olds, 0.6 for 4-year olds, and 0.8 for 3-year olds. ^b^ α_*g*_ = 1 for the Gibbs sampling stage and α_*g*_ = 0 for the particle filtering stage. ^c^ These values were used for the Gibbs sampling stage and were N/A for the particle filtering stage.									

**Figure A1 fig23:**
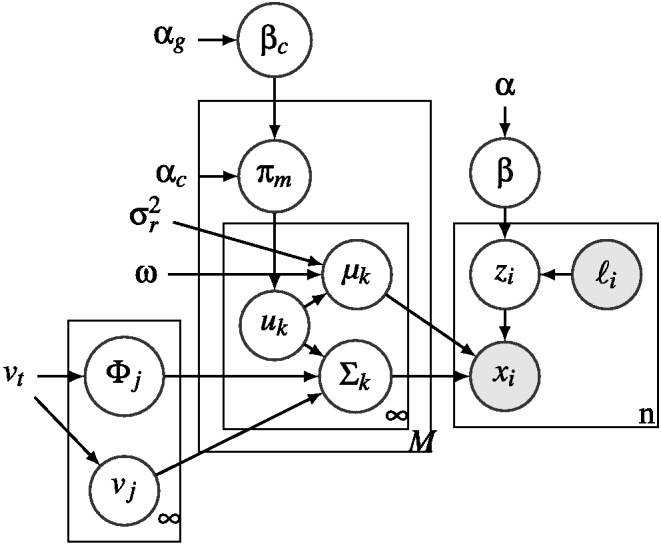
Plate Diagram Description of the Rational Exclusively Family RESemblance Hierarchy *Note*. Variables that are not circled are fixed, variables that appear in unshaded circles are unobserved, and variables that appear in shaded circles are observed.

## Footnotes

1 This formulation is often used in statistics (e.g., Neal, 1998), while Anderson (1991)’s formulation, which used a coupling parameter, can be recovered by setting $\alpha =\frac{1\;-\;\text{coupling}}{\text{coupling}}$.

2 To reduce complexity, we assume that there is a single shared prior for cluster means, though it is possible to extend the model to allow each component to have a different prior for cluster means.

3 Available at https://neicommons.nei.nih.gov/#/objectcolorstatistics

4 Hue deserves special consideration because it is a circular dimension, and so it may only be sensible to consider correlations in a local region of the space. We restricted the analysis to categories that had a standard deviation less than that of a uniform distribution (105 of the 107 total categories) and found the same pattern of correlations in variability between dimensions. We separately removed the 4% of images with hues that were close to the boundary (within π/4 of the edges of the 0–2π range) and also found the same pattern of correlations in variability between dimensions.

5 We minimized the sum of squared deviations across each point between REFRESH’s predictions and a transformation of the Minkowski distance metric *d* in Equation 1 into similarity: *s*_1_exp(−*s*_2_*d*). We found the best fitting weights, *w*_*i*_, exponent, *r*, and scaling parameters *s*_1_ and *s*_2_, which respectively scaled the range of similarity and how quickly it decreased depending on distance.

6 The probability of a stimulus located at the mean of a multivariate Gaussian distribution is (for a diagonal covariance matrix) proportional to the inverse of the product of the standard deviations. Assuming that all *c*_*d*_ = 1, for Ψ_*nww*_, Ψ_*wnw*_, and Ψ_*wwn*_ this is 10, while for Ψ_*wnn*_, Ψ_*nwn*_, and Ψ_*nnw*_ using smaller standard deviations that are 30% of the larger produces the similar value of 11.1. If we had made the smaller standard deviations of Ψ_*wnn*_, Ψ_*nwn*_, and Ψ_*nnw*_ 10% of the larger standard deviation, this value would have increased to the much larger 100.

7 Ensuring equal probability at the mean of the a multivariate Gaussian distribution requires that in Equation 1 there is a constant ∏_*i*_*w*_*i*_^1/2^, rather than the usual constraint of a constant ∑_*i*_*w*_*i*_.

8 Similar results can be obtained by assuming that older age groups have learned about a greater number of categories, as we showed in an earlier version of this work (Heller et al., 2009).

9 The untrained version of REFRESH incorrectly casts saturation and brightness as novel dimensions. However, the prior we use for integral dimensions in the trained model is effectively the same as for novel dimensions, except it cannot be influenced by the training data. As a compromise between these extremes, we modeled these results with more certainty in the initial isotropic prior: using either *v*_*t*_ = 100 or *v*_*t*_ = 1,000 instead of *v*_*t*_ = 30, because as *v*_*t*_ grows larger the prior is less influenced by the training data. All values of *v*_*t*_ produced the same qualitative results, except that if *v*_*t*_ = 1,000 the training advantage for the 2D Brightness and Saturation condition was not as large as in the 1D conditions.

## Appendix

Here we explain the link between multivariate Gaussian distributions and Euclidean similarity metrics, and give further details about the RMC, REFRESH, and how REFRESH is approximated in our simulations.

## The Link Between the Multivariate Gaussian Distribution and the Weighted Euclidean Metric

In the multivariate Gaussian distribution, the probability of a stimulus coming from a cluster is a monotonic transformation of its Mahalanobis distance from the mean of the cluster: *d* = ((*x* − μ)^*T*^Σ^−1^(*x* − μ))^0.5^, where *x* is a vector that describes the position of a stimulus in the space, μ is the mean of the cluster, and Σ is the covariance matrix of the cluster. If the covariance matrix only has diagonal elements, this can be rewritten as the sum *d* = (Σ_*i*_(1/σ_*i*_^2^)(*x*_*i*_ − μ_*i*_)^2^)^0.5^, where σ_*i*_^2^ is the *i*th diagonal element of Σ and *x*_*i*_ and μ_*i*_ are the values of these points on the *i*th dimension. This is now equal to the weighted Minkowski metric in Equation 1 when *r* = 2 and *w*_*i*_ = 1/σ_*i*_^2^.

However, if the covariance matrix of the multivariate Gaussian distribution has nondiagonal elements, then there is always a unique rotation matrix *R* that will transform the covariance matrix into a diagonal matrix Λ, with Σ = *R*Λ*R*^*T*^.

Using matrix operations, we can show that the rotation matrix can be rearranged so that its inverse applies to the dimensions of the representation of the stimulus *x* and the mean μ

$$d=\sqrt{{\left(x-\mu \right)}^{T}{\left(R\Lambda R^{T}\right)}^{-1}(x-\mu ))}\;=\sqrt{{\left(x-\mu \right)}^{T}{\left(R^{T}\right)}^{-1}\Lambda^{-1}R^{-1}(x-\mu ))}\;=\sqrt{{\left(R^{-1}(x-\mu )\right)}^{T}\Lambda^{-1}R^{-1}(x-\mu ))}\;=\sqrt{{\left(R^{-1}x-R^{-1}\mu \prime \right)}^{T}\Lambda^{-1}(R^{-1}x-R^{-1}\mu ))},$$

where in two dimensions

$$R^{-1}=(\begin{array}{cc} \cos(\theta ) & -\sin(\theta )\\ \sin(\theta ) & \cos(\theta ) \end{array}),$$

and this form can be generalized to additional dimensions.

There are two implications of this that are important for our purposes. First, this means that the multivariate Gaussian distribution is a generalization of the weighted Euclidean metric that encodes not just the weights but also the dimensions (i.e., which are a rotation of the original representing dimensions) along which the weights operate. Second, any set of original representing dimensions is equally good. This is because any rotation of the dimensions along which the covariance matrix is diagonal can be applied and the Mahalanobis distance remains unchanged. Essentially the representing dimensions are irrelevant for the probabilities of the multivariate Gaussian distribution.

A generalization of this point, which is important for REFRESH, is that the representing dimensions can also be made irrelevant for a covariance matrix Σ drawn from an inverse-Wishart (IW) distribution. First, we note that its probability density function depends on Σ and the covariance parameter Φ only through ∣Σ∣, ∣Φ∣, and tr(ΦΣ^−1^). ∣Σ∣ = ∣Λ∣ and so is unaffected by rotation, because ∣*R*Λ*R*^*T*^∣ = ∣*R*‖Λ‖*R*^*T*^∣ and ∣*R*∣ = ∣*R*^*T*^∣ = 1. Likewise, ∣Φ∣ = ∣*R*^*T*^Φ*R*∣. Because *R* is an orthogonal matrix (i.e., *R*^*T*^ = *R*^−1^) and because of the cyclical property of the matrix trace, tr(ΦΣ^−1^) = tr(Φ(*R*Λ*R*^*T*^) ^−1^) = tr(Φ*R*Λ^−1^*R*^*T*^) = tr((*R*^*T*^Φ*R*)Λ^−1^). That is, we can simply rotate the covariance parameter Φ′ = *R*^*T*^Φ*R* to allow the IW probability distribution to remain unchanged when rotating the representing dimensions for Σ.

The above arguments, however, become more complicated if a category is represented by multiple clusters (i.e., multivariate Gaussian distributions) as REFRESH can do. Except by design or exceptional luck, the covariance matrices of the different clusters will not be aligned, meaning that there is no single rotation of the axes of the space that can make every covariance matrix diagonal. If that is the case, then it will no longer be possible to interpret the category representation as a weighted Euclidean metric, as each cluster would imply a different weighted Euclidean metric. However, it remains the case that any set of representing dimensions is equally valid, as the Mahalanobis distances for each cluster will be unchanged by any rotation of the axes of the space.

## Details of the RMC

In the RMC (Anderson, 1991), the probability of each category that a new stimulus could belong to, given all of the previous stimuli and their labels is

$$P(l_{n}=i|x_{n},\;x_{n-1},\;l_{n-1})\propto P(l_{n}=i,\;x_{n}|x_{n-1},\;l_{n-1}),$$ A1

where *x*_*n*_ is the *n*th stimulus and *l*_**n**_ = *i* assigns that stimulus to category label *i*. The remaining stimuli are collected into the vector **x**_**n−1**_ and the known labels for these stimuli are collected in the vector *l*_**n − 1**_. While the right-hand side of Equation A1 is just the left-hand side rewritten, we show it here to emphasize how the RMC considers category labels to be dimensions of the stimuli like any other, in contrast to most other models of categorization and REFRESH.

When making the judgment about the label of a new stimulus, every possible assignment of stimuli to clusters is considered

$$P(l_{n}=i,\;x_{n}|x_{n-1},\;l_{n-1})=\sum_{z_{n}}P(l_{n}=i,\;x_{n}|z_{n},\;x_{n-1},\;l_{n-1})P(z_{n-1}|x_{n-1},\;l_{n-1})P(z_{n}|z_{n-1}),$$ A2

where *z*_*n*_ is the cluster index of the *n*th stimulus, **z**_**n**_ is a vector of the cluster indices of all of the stimuli, and **z**_**n−1**_ is a vector of the cluster indices of all previous stimuli. The CRP prior for the clusters, *P*(*z*_*n*_ ∣ **z**_**n−1**_), is given in Equation 2.

Inferring the assignments of stimuli to clusters can be done using essentially the same computation as on the right-hand side of Equation A2, but involving only the previous stimuli

$$P(z_{n-1}|x_{n-1},\;l_{n-1})\propto P(l_{n-1},\;x_{n-1}|z_{n-1},\;x_{n-2},\;l_{n-2})P(z_{n-2}|x_{n-2},\;l_{n-2})P(z_{n-1}|z_{n-2}),$$ A3

which shows how inferring the cluster indices can be done iteratively instead of all at once.

As a result of the assumed independence between the features of the items within a cluster,

$$P(l_{n}=i,\;x_{n}|z_{n},\;x_{n-1},\;l_{n-1})=P(l_{n}=i|z_{n},\;l_{n-1})P(x_{n}|z_{n},\;x_{n-1}).$$ A4

The probability of the label of the *n*th stimulus taking value *i*, *l*_*n*_ = *i*, then follows a beta-binomial distribution because the category labels are discrete features

$$P(l_{n}=i|l_{n-1},\;z_{n-1})=\frac{B_{i}+\beta }{B_{.}+2\beta },$$ A5

where *B*_*i*_ is the number of stimuli with label *i* within the same cluster, *B*_._ is the number of stimuli in the cluster, and β is a parameter.

For problems that are described with binary features, like the SHJ problems, the beta-binomial distribution is used for the likelihood of the perceptual features of the stimuli as well. However, for continuous stimuli like in the condensation versus filtration problems, the likelihood used for the perceptual features is Gaussian along each separable dimension

$$P(x_{n}|z_{n}=k,\;z_{n-1},\;x_{n-1})=\prod_{d}\int_{\mu_{k}^{\left(d\right)}}\int_{\Sigma_{k}^{\left(d\right)}}N(x_{n}^{\left(d\right)};\mu_{k}^{\left(d\right)},\;\Sigma_{k}^{\left(d\right)})P(\mu_{k}^{\left(d\right)}|\Sigma_{k}^{\left(d\right)},\;z_{n-1},\;x_{n-1})\,P(\Sigma_{k}^{\left(d\right)}|z_{n-1},\;x_{n-1})d\mu_{k}^{\left(d\right)}d\Sigma_{k}^{\left(d\right)},$$ A6

where the mean and variance of the Gaussian distribution for the *k*th cluster on the *d*th dimension is given by μ_*k*_^(*d*)^ and Σ_*k*_^(*d*)^ respectively, and the priors are given in Equation 3.

Finally, we explain here why the RMC for continuous data cannot produce violations of the triangle inequality, as demonstrated empirically with direct similarity judgments by Dunn (1983) and Tversky and Gati (1982). Once the priors over parameters are incorporated, the marginal distribution along each continuous dimension for the RMC is a Student’s *t* distribution and the dimensions are independent (Anderson, 1991). For any pair of dimensions, if the iso-probability curves were to have concavities, then this would mean that *P*(*x*) = *P*(*x*′) where *x* is closer to the *t*-distribution mean on both of the dimensions (or on at least one of the dimensions while remaining equal on the other) than *x*′. But this is impossible as *P*(*x*) = ∏_*d*_*P*(*x*^(*d*)^), *P*(*x*^′^) = ∏_*d*_*P*(*x*^′^^(*d*)^), and *P*(*x*^(*d*)^) > *P*(*x*′ ^(*d*)^) on both dimensions. (Or *P*(*x*^(*d*)^) > *P*(*x*′ ^(*d*)^) on one dimension and *P*(*x*^(*d*)^) = *P*(*x*′ ^(*d*)^) on the other.) Figure 5 demonstrates that concavities are necessary to produce violations of the triangle inequality, so if we take the iso-probability curves as iso-similarity curves, the RMC cannot produce violations of the triangle inequality. Even in the more complex similarity function of Equation 5, for the RMC similarity would be proportional to these probabilities, and thus the iso-similarity curves would be convex, and the triangle inequality would not be violated.

## Details of REFRESH

The MATLAB code used to implement REFRESH is available here: https://osf.io/fr7nq/.

In our initial formulation of REFRESH’s hierarchical prior we set the likelihood of a new stimulus given the other stimuli and cluster assignments to

$$P(x_{n}|z_{n}=k,\;z_{n-1},\;x_{n-1})=\int_{\mu_{k}}\int_{\Sigma_{k}}\int_{\Phi }P(x_{n}|\mu_{k},\;\Sigma_{k})P(\mu_{k}|z_{n-1},\;x_{n-1})P(\Sigma_{k}|\Phi ,\;z_{n-1},\;x_{n-1})\,P(\Phi )du_{k}d\Sigma_{k}d\Phi ,$$ A7

where we assume that the data *x*_*n*_, are distributed according to a multivariate Gaussian distribution with mean vector μ_*k*_ and covariance matrix Σ_*k*_. Each mean μ_*k*_ is independently drawn from a multivariate Gaussian distribution, μ_*k*_ ∼ *N*(ω, σ_*r*_^2^*I*), where σ_*r*_^2^*I* is an isotropic covariance matrix, meaning that σ_*r*_^2^*I* is not oriented toward any particular direction in the space. For the covariance matrix, the details are given in the main text. Comparing Equation A7 to Equation A6, we can see how the RMC likelihood has changed to accommodate a multivariate Gaussian distribution, the use of multivariate priors on the mean and covariance of each cluster, the addition of higher level priors for the priors on the covariance, and the breaking of the dependence between the mean and covariance of clusters.

In this initial formulation, we can think of the parameter Φ as providing a learned prior bias toward how clusters are oriented in the space of stimuli. The prior on Φ is set to be isotropic, meaning that the model begins with no particular orientation in the space. When Φ is learned from the data, it will then elongate along the dimensions in the space upon which the experienced clusters have high variance and contract along the dimensions in the space upon which the clusters have low variance. However, as we argue in our introduction of REFRESH above, in order to explain the dimensional biases, we need a more flexible formulation of the hierarchical prior. We do this in the same way that we provide flexibility to the clusters within a category: We turn this prior into an infinite mixture of different components. More formally, assuming that we know that cluster *k* is associated with mixture component *j*, we replace Equation A7 with

$$P(x_{n}|z_{n}=k,\;u_{k}=j,\;z_{n-1},\;u_{k-1},\;x_{n-1})=\int_{\mu_{k}}\int_{\Sigma_{k}}\int_{\Phi_{j}}\int_{v_{j}}P(x_{n}|\mu_{k},\;\Sigma_{k})P(\mu_{k}|z_{n-1},\;x_{n-1})P(\Sigma_{k}|\Phi_{j},\;z_{n-1},\;x_{n-1})\,P(\Phi_{j},\;v_{j}|z_{n-1},\;u_{k-1},\;x_{n-1})du_{k}d\Sigma_{k}d\Phi_{j}dv_{j}\text{,}$$ A8

where Φ_*j*_ is the covariance matrix parameter and *v*_*j*_ is the degrees of freedom of the *j*th component IW distribution in the mixture. The value *u*_*k*_ = *j* is the assignment of cluster *k* to mixture component *j*, which gives Σ_*k*_ an IW prior distribution with matrix parameter Φ_*j*_ and degrees of freedom *v*_*j*_. As with our first formulation of the hierarchical prior, we use another IW prior, but with an identity matrix for the mean parameter, for *P*(Φ_*j*_) so as not to bias the Φ_*j*_ components toward a particular set of dimensions. This isotropic prior on *P*(Φ_*j*_) was given degrees of freedom *v*_*t*_ and scale matrix *v*_*t*_^2^*I* so that the modal cluster covariance matrix was close to *I*. The prior on *v*_*j*_ was a truncated normal distribution that was truncated from below at the number of dimensions of the stimuli *D* because an IW distribution requires *v*_*j*_ > *D* − 1. The mean and standard deviation of the prior were both set equal to *v*_*t*_ so as to bias the value learned from the data toward *v*_*t*_.

The CRP prior on the assignments **z** is given in Equation 2, while the hierarchical CRP prior on the assignments **u** is a two-stage elaboration of this process. In the first stage, components were chosen proportional to the number of clusters in the current context that were assigned to each component. If a “new” component was chosen to introduce into the current context, then in the second stage a component was chosen proportional to global number of clusters assigned to each component. If a new component was chosen in this second stage as well, the cluster was assigned to a completely new component,

$$P(u_{k}=j|u_{-k})=\{\begin{array}{cc} \frac{C_{j}}{n_{c}\;-\;1\;+\;\alpha_{c}}+\frac{\alpha_{c}G_{j}}{\left(n_{c}\;-\;1\;+\;\alpha_{c})(n_{g}\;-\;1\;+\;\alpha_{g}\right)} & \text{if}\;\;G_{j}>0(\text{i.e.},\;j\;\text{is old})\\ \frac{\alpha_{c}\alpha_{g}}{\left(n_{c}\;-\;1\;+\;\alpha_{c})(n_{g}\;-\;1\;+\;\alpha_{g}\right)} & \text{if}\;\;G_{j}=0\;\;(\text{i.e.},\;j\;\text{is new}), \end{array}$$ A9

where **u**_−**k**_ is all component assignments **u** except for the *k*th, *n*_*c*_ is the number of clusters in the current context, *C*_*j*_ is the count of clusters associated with component *j* in the current context, *n*_*g*_ is the count of all clusters across contexts, *G*_*j*_ is the count of clusters across all contexts that are assigned to component *j*, α_*c*_ is the dispersion parameter for the current context, and α_*g*_ is the global dispersion parameter.

Figure A1 shows a plate diagram of REFRESH. The variables β, β_*c*_, and π_*m*_ are distributed as GEM(α), GEM(α_*g*_), and GEM(α_*c*_) respectively (where GEM stands for Griffiths, Engen, and McCloskey, Pitman (1996)), which are stick-breaking distributions that are formulated sequentially in Equations 2 and A9. *M* is the number of contexts. Table A1 shows the parameterized covariance matrices used in the trained REFRESH approximation.[Fig-anchor fig23] [Table-anchor tbl3]

## Approximating REFRESH

To simulate REFRESH results, we used four different approximations, choosing the approximation appropriate for each task we simulated: Exact calculation of partition probabilities from the approximate trained model, particle filter simulations of incremental category learning, a Gibbs sampler approximation of component learning, and finally Gibbs sampling followed by particle filtering.

## Exact Partition Probabilities From the Approximate Trained REFRESH

The first kind of approximation assumed that Φ_*j*_ was fixed for each component as the result of training on real-world categories, then the probability of partitioning the stimuli into different categories was calculated exactly. This approximation was used only for the simulation for the Smith (1989) experiment as it contained only six stimuli, so it was tractable to calculate the probabilities of the 203 possible partitions of the stimuli.

## Particle Filter Simulations of Incremental Category Learning Using the Approximate Trained REFRESH

Most of the simulations using the trained version of REFRESH called for the model to produce the probability of category labels incrementally as a sequence of stimuli were presented to the model. For these simulations with the trained REFRESH, we used a particle filter algorithm with 100 particles. Each particle in the particle filter contained a complete and fixed assignment of the already-observed stimuli to clusters, and an assignment of all of these clusters to components. When a new stimulus was observed, the probability of its category label was calculated for each particle and the label probability was averaged over the particles. When category feedback was given, the representation was updated by calculating for each particle the probability of this new stimulus’ assignment to each cluster within the same category (including a new cluster, using a component assignment sampled from the prior probabilities over components). In effect these probabilities were then pooled across particles, and a new set of 100 particles was sampled with replacement from this pool. Each particle filter simulation was repeated 10 times and the outputs were averaged to produce REFRESH simulation results, which we found to be stable.

## Gibbs Sampling of Component Learning in the Full REFRESH

Finally, Gibbs sampling was used for simulations using the full REFRESH that investigated how the components were learned and updated. We sampled the parameters iteratively given both the training data and the sampled values of the other sets of parameters. First, we iteratively sampled the cluster means μ_*k*_ and cluster covariances Σ_*k*_ for each cluster *k*. Before any data were observed, the prior distributions of the cluster means and covariances were independent,

$$\mu_{k}|u_{k}=j\;∼\;N(\omega ,\;\sigma_{r}^{2}I)\Sigma_{k}|u_{k}=j\;∼\;IW_{v_{j}}(\Phi_{j}),$$ A10

where ω was fixed to the mean value of the stimuli. However, once a set of *n*_*k*_ observations *X* are assigned to cluster *k*, these variables are no longer independent. In this case, we performed several Gibbs sampling steps iterating between these two variables alone before moving to the next cluster,

$$\mu_{k}|u_{k}=j,\;X\;∼\;N(\Sigma_{k}^{\prime }(n_{k}\Sigma^{-1}\bar{X}+{\left(\sigma_{r}^{2}I\right)}^{-1}\omega ),\;\Sigma_{k}^{\prime })\Sigma_{k}|u_{k}=j,\;X\;∼\;IW_{v_{j}+n_{k}}(\Phi_{j}+(X-\mu_{k})(X-\mu_{k}{)}^{T}),$$ A11

where Σ_*k*_^′^ = ((σ_*r*_^2^***I***)^−1^ + *n*Σ_*k*_^−1^)^−1^ and $\bar{X}$ is the mean of the observations *X*.

Next, the cluster membership for each observation *z*_*i*_ was sampled given the cluster assignments of each other observation **z**_−**i**_ and the cluster means and cluster covariances. The probability of each assignment was proportional to the prior on each cluster is given by Equation 2, multiplied by the likelihood of an observation *x* being associated with cluster *k*, which was given by a multivariate normal distribution with mean μ_*k*_ and covariance Σ_*k*_. A similar process was used to sample component membership for each cluster. The likelihood that a cluster was assigned to a component was calculated using Equation A10.

Finally, the parameters associated with each component were sampled. We sampled each component covariance Φ_*j*_ and degrees of freedom *v*_*j*_ jointly using the Metropolis–Hastings algorithm. Beginning with the current values of a component covariance Φ_*j*_ and degrees of freedom *v*_*j*_, we iteratively proposed a new value using an IW proposal distribution for the covariance: IW_*vt*_(*v*_*t*_ Φ_*j*_). The mean of this proposal distribution was scaled up by the degrees of freedom so that the proposed covariance matrix was on average of a similar magnitude to the state covariance matrix. New values of *v*_*j*_ were proposed from a normal distribution, *N*(*v_j_*, *v*_*t*_^2^), centered on the current value. The posterior probability of each pair Φ_*j*_ and *v*_*j*_ was proportional to the product of the priors, *p*(Φ_*j*_) = IW_*vt*_(*I*) and *p*(*v_j_*) = TN(*v_t_*, *v*_*t*_^2^), multiplied by the product of the likelihood given in Equation A10. In each step of the Metropolis–Hastings algorithm, the acceptance probability was given by the usual Metropolis rule, which corrected for asymmetries in the proposal distributions.

## Gibbs Sampling Then Particle Filtering

For one simulation (see Table A2), in order to observe the effect of categorization training on later category learning performance, we first used Gibbs sampling to produce samples of the components from the full version of REFRESH as a result of training. Then for each sample, we extracted a set of fixed covariance matrices, one covariance matrix for each component in that sample, with each covariance matrix being the mode of the IW distribution associated with that component. For each sampled set of fixed covariance matrices, we then ran the particle filter approximation to show the effect of the trained components on later category learning performance, and averaged performance over samples. While it was necessary to do this for computational tractability, this is a very approximate procedure as it removed the full version of REFRESH’s uncertainty about the components in the later category learning. Averaging over the predictions from each sample may not produce the same results as using the full set of samples to make each prediction.[Table-anchor tbl4]

## Discussion of REFRESH Parameter Values

We present a summary of the parameters used in each simulation of REFRESH in Table A2. We were not able to conduct a formal sensitivity analysis of how the results REFRESH produces change depending on different parameter values, due both to the computational complexity of the model and to the number of results that would have to be produced. However, here we informally discuss our observations as to how the simulation results change in response to changes in the parameters.

As Table A2 shows, *c*_*d*_ was the least consistent parameter across simulations. This parameter scales the standard deviations of the covariance matrices assumed in the particle filter approximation. Large values of this parameter can be thought of as shrinking the psychological space while small values stretch it, and this stretching or shrinking influences how likely it is that REFRESH produces large or small clusters of stimuli. Tuning this parameter was necessary to produce performance of the same level as human participants. Ideally this parameter would be held constant and only the values of the stimuli along each dimension would vary, but stimulus values often vary substantially across experiments, so we view this parameter as compensating for that variability. An exception to this general rule is the biconditional discrimination experiment of Soto et al. (2015) which controlled stimuli values across integral and separable dimensions, and for this simulation we held *c*_*d*_ constant across integral and separable dimensions.

While the covariances chosen for the particle filter approximation differ from simulation to simulation, these were chosen for principled reasons as discussed in the main text in the description of each simulation.

The parameters α_*g*_, *v*_*t*_, and σ_*r*_^2^ varied according to the approximation used. The particle filter approximation used a fixed number of covariance components, requiring α_*g*_ = 0, and the Gibbs sampler approximation inferred the number of covariance components, requiring α_*g*_ > 0. Larger values of α_*g*_ would make it more likely for the model to infer more (perhaps redundant) components. *v*_*t*_ determines how “strong” the isotropic prior is, with larger values likely requiring REFRESH to receive more training before producing separable dimension iso-similarity curves. σ_*r*_^2^ determines how much the mean of each cluster is pulled toward a fixed central value. σ_*r*_^2^ = 1 was used with the Gibbs sampler approximations to allow components to be formed in response to stimuli across the entire stimulus space, while σ_*r*_^2^ = 0.1 was used with the particle filter approximation in order to slow the REFRESH’s rate of learning new categories. One hundred particles were used to approximate REFRESH as a computational-level model, but a more psychologically plausible number of particles (see Rational Process Models section) should slow learning to human levels, and we anticipate that a consistent value of σ_*r*_^2^ could then be used.

α and α_*c*_ were almost always consistent across simulations. Smaller values of α would result in smaller numbers of clusters being inferred, and this relatively high value was important for ensuring that the Gibbs sampler approximation did not learn one cluster for all training stimuli and thus one component overall. Larger values of α_*c*_ would allow a better chance of multiple components being used in the same context, and we show in the main text (see Figure 14B) that this would prevent REFRESH from learning SHJ Type II categories faster than Type III categories.

Unique parameter values for α and σ_*r*_^2^ were used in the simulation of the Smith (1989) experiment. While in all of the other simulations the trained model was predicting binary responses, in this simulation it was predicting which of 203 possible responses REFRESH would make, 198 of which would be marked as the uninteresting “other.” The standard values of α and σ_*r*_^2^ caused almost all predictions to be “other,” so we used these unique settings to better understand the developmental trajectory of the REFRESH’s predictions.
